# Healthcare Resource Utilization and Cost After Temperature‐Controlled Radiofrequency Treatment of Nasal Airway Obstruction: A Real‐World Longitudinal Claims Analysis

**DOI:** 10.1002/alr.70066

**Published:** 2025-11-26

**Authors:** David W. Kennedy, Gavin Setzen, Ashleigh A. Halderman, Kevin C. Welch, Bobby Tajudeen, Gary M. Owens, Paul J. Niklewski, Masayoshi Takashima

**Affiliations:** ^1^ Sidney Kimmel Medical College Thomas Jefferson University Philadelphia Pennsylvania USA; ^2^ Department of Otolaryngology Head and Neck Surgery Albany Medical College, Albany ENT & Allergy Albany New York USA; ^3^ Department of Otolaryngology Head and Neck Surgery Westchester Medical Center Health Network Valhalla New York USA; ^4^ Department of Otolaryngology Head and Neck Surgery Northwestern University Feinberg School of Medicine Chicago Illinois USA; ^5^ Department of Otolaryngology‐Head and Neck Surgery Rush University Medical Center Chicago Illinois USA; ^6^ Gary Owens Associates Ocean View Delaware USA; ^7^ Department of Pharmacology and Systems Physiology College of Medicine University of Cincinnati Cincinnati Ohio USA; ^8^ Department of Otolaryngology—Head & Neck Surgery Houston Methodist Hospital Houston Texas USA

**Keywords:** cost‐effectiveness, healthcare resource utilization, minimally invasive surgery, nasal airway obstruction, nasal valve collapse, temperature‐controlled radiofrequency

## Abstract

**Background:**

Nasal airway obstruction (NAO) is prevalent with substantial health and quality of life burdens. Nasal valve collapse (NVC) is one structural cause of NAO. Temperature‐controlled radiofrequency (TCRF) nasal valve remodeling offers an alternative to invasive surgery. Clinical efficacy is established, but the impacts of TCRF on healthcare resource utilization (HRU) and cost in real‐world settings remain underexplored.

**Methods:**

Two cohorts with NAO were defined from a large general NAO population: the TCRF cohort with an isolated TCRF (index) procedure and a propensity‐matched medically managed (MM) cohort without nasal procedures. HRU and costs were evaluated within a 24‐month pre‐/post‐index period for both.

**Results:**

A total of 10,206 TCRF and 50,766 MM patients were analyzed. Significant post‐index reductions were observed for TCRF across all‐cause Evaluation & Management (E&M) visits, ENT‐related procedures, and sleep‐related claim categories. A large reduction in mean daily post‐index costs was seen for the TCRF cohort: $68.07 pre‐index to $38.75 post‐index (−43.1%). Mean daily costs went up in the MM cohort from $42.08 pre‐index to $63.26 post‐index (+50.4%), resulting in total cost savings of $21,418.26 for the TCRF cohort and a total cost increase of $15,471.99 in the MM cohort in the 24‐month post‐index period. TCRF cost reductions were driven by reductions in NAO‐related HRU.

**Conclusions:**

In this large, real‐world analysis, TCRF treatment for NVC‐related NAO demonstrated substantial reductions in HRU and total costs of care, demonstrating sustained savings over 2 years relative to MM patients.

## 1 Introduction

Nasal airway obstruction (NAO) is a highly prevalent, underappreciated condition that affects millions of people worldwide [1]. NAO is associated with a substantially negative impact on quality of life (QOL), with afflicted patients experiencing decrements in health utility on par with common chronic disorders such as renal disease, Type 2 diabetes, and chronic obstructive airway disease [2, 3]. NAO is associated with sleep disturbances, cognitive impairment, and insomnia [4]. There is a direct relationship between increased nasal resistance secondary to nasal obstruction and sleep‐disordered breathing, impacting sleep physiology and architecture [5, 6], QOL [4, 7, 8], and presenteeism at work [9]. Successful treatment of NAO can be profoundly beneficial by restoring normal breathing, reducing fatigue, improving sleep, and enhancing daily function [4, 10, 11]. Exemplifying this, nasal interventions to restore nasal patency have been associated with higher CPAP compliance and lower pressures in patients with co‐morbid obstructive sleep apnea [12, 13, 14].

Nasal valve collapse (NVC), a structural cause of NAO, has traditionally been addressed with surgical repair of the nasal valve [15, 16]. A minimally invasive option using temperature‐controlled radiofrequency (TCRF) energy to remodel and strengthen the nasal valve cartilage is an alternative to traditional surgery (VivAer, Aerin Medical, Mountain View, CA) that can be performed in an office setting. TCRF restores nasal patency, decreasing nasal airflow resistance in this narrowest part of the nasal airway [16]. Long‐term safety and efficacy have been consistently demonstrated in multiple clinical studies of TCRF treatment of NVC‐related NAO [11, 17, 18, 19].

Minimally invasive surgical (MIS) approaches reduce recovery time, exposure to surgical risks (e.g., general anesthesia, post‐operative infections, adverse events), and can be associated with faster time to recovery, reduced pain medication utilization, and reduced work absenteeism [20]. Healthcare spending in the US continues to rise, with health expenditures increasing by 7.5% to $4.9 trillion in 2023 [21], outpacing GDP growth, with costs anticipated to account for 20.1% of US GDP in 2025. Ideally, the introduction of new technologies into the healthcare system should drive measurable improvements, making patients healthier and the healthcare system more efficient so that the most value is realized for every healthcare dollar spent.

Clinical trials are essential to establish the baseline characteristics of device performance, but can be practically constrained. Real‐world data (RWD) collected during routine clinical care can provide new insights and support robust evaluations of clinical performance, outcomes, and costs associated with device usage within the healthcare system. Real‐world evidence (RWE) leveraging high‐quality aggregated RWD, such as those from large claims databases, enables evaluation of a wider population and analysis of device exposures and outcomes of interest on a scale impractical for clinical trials.

This is the first study using RWD evaluating the impact of TCRF as a minimally invasive option for the treatment of NVC‐related NAO. The primary objective of this study was to evaluate and quantify healthcare resource utilization (HRU) and cost trends associated with TCRF nasal valve remodeling for NVC‐related NAO in the general US population.

## 2 Methods

### 2.1 Study Design and Patient Population

Using a large, de‐identified, patient‐level claims database (The Komodo Healthcare Map, NY, NY), this longitudinal, retrospective study evaluated patients with NAO who underwent an isolated TCRF between January 2023 and June 2025. A general NAO population with continuous enrollment was derived using NAO‐specific ICD‐10‐CM diagnostic codes (Table 1) from which two cohorts were derived: (1) TCRF cohort (TCRF) consisting of patients undergoing a TCRF nasal valve repair (CPT 30469) without other nasal procedures within ± 2 days of the TCRF procedure; and (2) NAO medically managed (MM) cohort, consisting of propensity‐matched MM NAO patients without nasal procedures in the evaluation window with the same data availability in the 24‐month pre‐/post‐index period. Details of the propensity weighting/matching approach are provided in the Supporting Information. The ± 2‐day index window was chosen as a standard to capture TCRF‐associated claims. The primary analysis was performed on patients with claims within ± 24 months on either side of the index.

**TABLE 1:** CPT and diagnostic codes used to define the study population.

NAO diagnostic codes
J34.2 Deviated nasal septum
J34.3 Hypertrophy of nasal turbinates
J34.82 Nasal valve collapse
J34.89 Other disorders of the nose or nasal sinuses
J34.9 Unspecified disorder of the nose and nasal sinuses
J34.820^a^ Internal nasal valve collapse
J34.8200 Internal nasal valve collapse, unspecified
J34.8201 Internal nasal valve collapse, static
J34.8202 Internal nasal valve collapse, dynamic
J34.829 Nasal valve collapse, unspecified
M95.0 Acquired deformity of nose
R09.81 Nasal congestion

CPT codes for excluded concomitant nasal procedures
00160 Anesthesia procedures on the nose and accessory sinuses
30130 Excision inferior turbinate, partial or complete, any method
30140 Submucous resection inferior turbinate, partial or complete
31240 Nasal/sinus endoscopy, surgical; with concha bullosa resection
31242 Nasal/sinus endoscopy, surgical; with destruction by radiofrequency ablation, posterior nasal nerve
30520 Septoplasty/submucosal resection, with or without cartilage scoring, contouring, or replacement with graft
31254 Nasal/sinus endoscopy, surgical with ethmoidectomy; partial (anterior)
31256 Nasal/sinus endoscopy, surgical with maxillary antrostomy
31267 Nasal/sinus endoscopy, surgical with maxillary antrostomy and removal of tissue from maxillary sinus
31298^b^ Nasal/sinus endoscopy, surgical, with dilation (e.g., balloon dilation), frontal and sphenoid ostia

^a^ NVC codes not available until October 1, 2024.

^b^ Combined code for 31295–31297; unrelated ENT procedures (61782: stereotactic navigation, 69706: eustachian tube balloon dilation, 42826: tonsillectomy/adenoidectomy).

### 2.2 Comorbidity Scoring

Diagnosis codes were used to map to comorbidity categories using validated ICD‐10‐CM algorithms. For each TCRF patient, age, sex, pre‐index cost (in the 365 days prior to the procedure), and comorbidity burden were derived using propensity Charlson Comorbidity Index (CCI) scores. Equivalent variables were constructed for the MM cohort.

### 2.3 Nasal Airway‐Related HRU

All‐cause Evaluation and Management (E&M) visits were identified using grouped CPT codes. Common ENT‐related procedures were identified using relevant CPT codes. Nasal‐related prescriptions were captured from pharmacy claims using therapeutic class codes and generic drug names for nasal sprays.

For each outcome, rates/100 person‐years (PY) and exposure‐adjusted claims were calculated for the pre‐/post‐index periods, and post:pre rate ratios (RRs) were derived. Medication rates were standardized per 100 PY to account for unequal follow‐up between the cohorts. Because early postoperative withholding of nasal sprays could transiently affect fills, the persistence of reductions over time was examined using a time‐to‐discontinuation analysis (Figure 2). Prevalence was expressed as the proportion of patients with ≥ 1 claim in each period for procedural measures; any‐use percentages were calculated for pharmacy classes.

### 2.4 Sleep‐Related HRU

Sleep‐related HRU included sleep studies, surgery, and medications, with claims counted to reflect utilization burden; these measures are sensitive to refill patterns and supply cadence. Sleep‐related utilization codes are provided in Table S1. Sleep‐related outcomes were standardized per 100 PY using covered‐day denominators to ensure comparability among patients with variable observation time. Continuous‐enrollment data were used on either side of the index to minimize potential bias from limited follow‐up.

### 2.5 Time to Change in Healthcare Utilization

Pharmacy claims were predefined terms for nasal spray medications. For each patient, the last post‐index fill date was identified. Patients whose last fill occurred within 60 days of their coverage end date were censored, reflecting possible ongoing use. A Kaplan–Meier type survival curve was constructed, with the event defined as discontinuation of nasal spray use.

Monthly visit rates were expressed as visits/100 PY, and a 3‐month rolling average was applied to smooth short‐term fluctuations.

### 2.6 Revision Rates

NVC‐related revision procedures were identified using CPT codes for repeat TCRF (30469), functional rhinoplasty (30465, 30420, and 30400), and LATERA (Stryker, Kalamazoo, MI) absorbable nasal implant (30468) performed less than 2 days after the index date, with type classified based on the first revision CPT code.

### 2.7 Cost Outcomes

Cost outcomes were derived from allowed amounts across inpatient, outpatient, and pharmacy claims. For each patient, a total cost within the pre‐index (−730 to −3 days), index (−2 to +2 days), and post‐index (+3 to +730 days) windows was calculated and normalized to daily cost, dividing total cost by the number of in‐window coverage days. Days with coverage but no claims contribute zero cost to the average; days without coverage were excluded. Cost outcomes are presented in USD. To evaluate sensitivity, mean daily, mean monthly, and standardized 24‑month costs were re‐estimated after trimming the top and bottom 1% and 2% of patient‑level cost distributions (Tables S10 and S11).

### 2.8 Statistical Analysis

The primary analysis was a within‐patient pre‐/post‐comparison around the index procedure. HRU outcomes were standardized for unequal follow‐up by converting exposure to covered days from eligibility files and expressing results as events per 100 PY. Utilization was measured over 24‐month windows before/after the index procedure, excluding the ± 2‐day peri‐index period. A sensitivity analysis was performed to assess the impact of variable follow‐up times in the TCRF cohort.

Pre/post differences were assessed with paired tests appropriate to outcome type: McNemar tests for any‐use proportions and Poisson rate models for event rates, reported as RRs with 95% confidence intervals (CIs) and standard small‐cell corrections. Due to the large size of the cohorts, practical significance to assess similarities between groups was calculated using two one‐sided equivalence tests (TOST) with significance set at *p* < 0.05. Between‐cohort summaries are presented as weighted means/rates and RRs for context; the pre/post comparison is the primary analysis.

Statistical methods were aligned with each outcome type as follows: any‐use prevalence was assessed using McNemar tests; event rates were evaluated with patient‐level Poisson models (including log‐exposure offsets) to estimate RRs and 95% CIs; cost outcomes were summarized as exposure‐normalized means with non‐parametric bootstrap CIs; time‐to‐discontinuation of nasal sprays was analyzed using a Kaplan–Meier type estimator with censoring within 60 days of coverage end; and revision rates were described using cumulative incidence based on the first revision CPT code.

Poisson models were fit at the patient level with log‐exposure offsets and cluster‐robust (patient‐level) standard errors. Model dispersion was examined descriptively to assess potential over‐dispersion; Poisson specifications were retained as results were stable and consistent across outcomes.

Cost CIs were obtained via bias‐corrected and accelerated (BCa) bootstrap with 2000 replicates; percentile intervals are provided in sensitivity tables for comparison.

Analyses were performed in R (v4.5.1; http://www.r‐project.org). Details for all methods used are provided in the Supporting Information.

## 3 Results

### 3.1 Patient Population

The TCRF (*N *= 10,206; mean age 60.9 years) and MM (*N *= 50,766; mean age 60.1 years) cohorts were older than the general NAO population (*N *= 27,010,537; mean age 43.6 years) but statistically equivalent to each other. Between‐cohort comparisons used TOST with prespecified equivalence margins (age ± 3.38 years; categorical variables ± 10 percentage points). The higher mean age for the TCRF cohort could be expected for patients undergoing a nasal‐valve procedure. Comorbidities, as measured by the CCI, were also higher in the TCRF and MM Cohorts compared to the general NAO population, but statistically equivalent to each other. Sex distribution was similar between cohorts (Table 2).

**TABLE 2:** Baseline patient characteristics and insurance distribution by cohort.

Characteristic	General NAO population	MM cohort^a^ (weighted)	TCRF cohort	TCRF vs. MM cohort equivalence^b^ (% points^c^, *p*)
*N* ^b^	27,010,537	50,766	10,206	—
Age, mean (SD)	43.6 (24.1) CI: 43.6%–43.6%	60.1 (16.4) CI: 60.0%–60.2%	60.9 (17.4) CI: 60.6%–61.2%	Equivalent (± 3.38 yrs, *p* < 0.001)
**Gender, *n* (%)**				
Female	15,181,617 (56.2%) CI: 56.1%–56.3%	23,165 (45.6%) CI: 40.1%–51.2%	4761 (46.6%) CI: 45.7%–47.5%	Equivalent (± 10.0 pp, *p* < 0.001)
Male	11,556,121 (42.8%) CI: 42.7%–43.0%	27,601 (54.4%) CI:48.9%–59.9%	5297 (51.9%) CI: 50.9%–52.8%	Equivalent (± 10.0 pp, *p* < 0.001)
Unknown/other	272,799 (1.0%) CI: 1.0%–1.0%	0 (0.0%) CI: NA	148 (1.5%) CI: 1.2%–1.7%	Equivalent (± 10.0 pp, *p* < 0.019)
**Charlson Comorbidity Index**				
Charlson score, mean (SD)	1.5 (1.9) CI: 1.5%–1.5%	1.9 (2.1) CI: 1.9%–1.9%	2.2 (2.1) CI: 2.2%–2.2%	Equivalent (± 10.0 pp, *p* < 0.001)
Charlson score ≥ 1, %	60.9% CI: 60.9%–60.9%	65.1% CI: 64.7%–65.5%	74.7% CI: 73.9%–75.5%	Equivalent (± 10.0 pp, *p* < 0.001)
**Insurance distribution**				
Commercial	13,740,260 (50.9%) CI: 50.9%–50.9%	27,896 (55.0%) CI: 54.5%–55.4%	4016 (39.4%) CI: 38.4%–40.3%	Not equivalent (± 10.0 pp, *p* = 1.000)
Medicare	4,335,191 (16.1%) CI: 16.0%–16.1%	17,230 (33.9%) CI: 33.5%–34.4%	4364 (42.8%) CI: 41.8%–43.7%	Equivalent (± 9.7 pp, *p *= 0.045)
Medicaid	6,428,508 (23.8%) CI: 23.8%–23.8%	3447 (6.8%) CI: 6.6%–7.0%	952 (9.3%) CI: 8.8%–9.9%	Equivalent (± 5.4 pp, *p *< 0.001)
Unknown	2,506,578 (9.3%) CI: 9.3%–9.3%	2198 (4.3%) CI: 4.2%–4.5%	874 (8.6%) CI: 8.0%–9.1%	Equivalent (± 4.2 pp, *p* = 0.010)

Abbreviations: CI, 95% confidence interval; NA, not applicable; pp, percentage points.

^a^ NAO medical management cohort with no surgical interventions, propensity matched with average treatment effect weighting using age, sex, baseline comorbidity burden, and pre‐index healthcare costs.

^b^ Equivalence determined using two one‐sided alpha testing (TOST) method with significance for *p* < 0.05.

^c^ Unique patients.

Insurance distribution was equivalent across groups except for commercial coverage, which exceeded the prespecified margin of ± 10 percentage points (difference of −15.6 percentage points [95% CI: −16.6 to −14.6]; *p_*TOST = 1.000), reflecting current market coverage patterns.

Taken together, the isolated episode definition (CPT 30469 ± 2‐day window without concurrent procedures), similar sex distribution, equivalence for age and CCI scores, and low unknown category proportions compared to the MM cohort support the TCRF cohort as an appropriate base population for outcomes analyses while limiting confounding from concomitant procedures.

### 3.2 Nasal Airway‐Related HRU

The TCRF cohort had higher rates of nasal airway‐related HRU for every utilization type compared to MM (Table 3). However, nasal airway‐related HRU was meaningfully reduced following TCRF. E&M visit patterns for TCRF revealed marked reductions in several outpatient categories post‐index. Office/outpatient encounters decreased 31.0% (95,820 pre‐ to 66,106 post‐index in annualized claims), preventive (e.g., immunizations) visits declined 35.0% (3746 to 2436 claims), Telemedicine/Remote visits fell 39.2% (1705 to 1037 claims), and emergency department visits decreased 26.5% (7083 to 5203 claims), and home/residence visits decreased 14.4% (367 to 314 claims). Conversely, in the TCRF cohort, a 75.6% increase was seen in critical care (e.g., intensive care unit) visits (356 to 618 claims), a 17.3% increase in hospital inpatient/observation stays (4921 to 5772 claims), and a 13.8% increase in nursing facility visits (487 to 554 claims). The MM cohort had significant increases in all E&M visits except for preventive care. Notably, the magnitude of the increases was higher than those experienced by the TCRF cohort for critical care, hospital inpatient/observation, and nursing facility visits (Table 3). For interpretability, annualized pre‑ and post‑index claim counts are reported alongside rates in Table 3. Because the MM cohort was approximately five times larger than the TCRF cohort and absolute claim counts are correspondingly higher, rates per 100 PY are provided to enable appropriate per‐patient comparisons.

**TABLE 3:** Nasal airway‐related resource utilization pre‐ and post‐index: TCRF versus medically managed cohort.^a^

Resource utilization type	Rate/100 PY^b^ (pre)		Rate/100 PY^b^ (post)	Claims (pre)	Claims (post)	Rate ratios (95% CI), *p*
Office/outpatient	TCRF	3076.13	2726.95	95,820	66,106	0.89 (0.88–0.89), *p *< 0.001
	MM	913.55	988.3	927,670	1,003,217	1.08 (1.08‐1.09), *p *< 0.001
Emergency department	TCRF	227.63	196.62	7083	5203	0.86 (0.85–0.88), *p *< 0.001
	MM	107.36	118.3	108,981	120,192	1.10 (1.09–1.11), *p *< 0.001
Hospital inpatient/ observation	TCRF	158.17	218.3	4921	5772	1.38 (1.35–1.41), *p* < 0.001
	MM	105.65	188.81	107,223	191,527	1.79 (1.77–1.8), p < 0.001
Preventive care	TCRF	120.59	100.6	3746	2436	0.83 (0.81–0.86), *p* < 0.001
	MM	51.75	47.45	52,523	48,184	0.92 (0.9–0.93), *p* < 0.001
Telemedicine /remote	TCRF	55.0	42.91	1705	1037	0.78 (0.75–0.81), *p* < 0.001
	MM	16.28	21.65	16534	22,001	1.33 (1.3–1.36), *p* < 0.001
Nursing facility	TCRF	15.65	20.93	487	554	1.34 (1.25–1.43), *p* < 0.001
	MM	13.95	29.4	14,167	29,883	2.11 (2.06–2.16), *p* < 0.001
Home/ residence	TCRF	11.78	11.84	367	314	1.01 (0.928–1.089), *p* = 0.901
	MM	4.88	7.83	4956	7952	1.60 (1.54–1.67), *p* < 0.001
Critical care	TCRF	11.31	23.38	356	618	2.07 (1.97–2.218), *p *< 0.001
	MM	6.94	15.13	7049	15,376	2.18 (2.11–2.26), *p* < 0.001

ENT procedure type	Prevalence^c^ (%) (pre)	Prevalence^c^ (%) (post)	Rate/100 PY (pre)	Claims (pre)	Claims (post)	Rate/100 PY (post)	Rate ratio (95% CI), *p*
Nasal endoscopy	26.63	11.78	67.62	2189	1250	47.33	0.70 (0.68–0.73), *p *< 0.001
FESS	4.54	1.52	11.78	2404	731	3.58	0.30 (0.28–0.33), *p *< 0.001
Turbinate resection	4.24	1.29	13.92	451	159	6.04	0.43 (0.40–0.48), *p *< 0.001
Septoplasty	3.16	0.95	8.43	273	101	3.84	0.46 (0.40–0.51), *p *< 0.001
Balloon sinuplasty	1.37	0.65	4.33	135	82	3.1	0.72 (0.62–0.83), *p *< 0.001

Medication class ^d^	Cohort	Fills/100 PY (pre)	Fills/100 PY (post)	Claims (pre)	Claims (post)	Rate ratio (95% CI), *p*
Corticosteroid nasal sprays (glucocorticoid)	TCRF	87.04	81.67	9564	7276	0.94 (0.91–0.96), *p *< 0.001
	MM	50.00	68.69	50,766	69,737	1.37 (1.36–1.39), *p *< 0.001
Nasal antihistamine/ decongestant, including combination nasal sprays	TCRF	47.61	46.70	7084	5201	0.98 (0.95–1.02), *p *= 0.306
	MM	12.52	25.42	12,717	25,833	2.03 (1.98–2.08), *p *< 0.001
Combination steroid‐antihistamine nasal sprays	TCRF	6.46	9.69	709	933	1.50 (1.37–1.64) *p *< 0.001
	MM	2.01	3.44	2041	3495	1.71 (1.61–1.83), *p *< 0.001

^a^ NAO MM Cohort had ENT procedures excluded to reflect medical management only.

^b^ Rates are expressed in 100 patient‐years, adjusted for individual follow‐up time; rate ratios with 95% confidence intervals are derived from Poisson approximation with *p* values from exact two‐sample Poisson tests. A two‐sided *p* < 0.05 is considered significant.

^c^ Prevalence is calculated as the proportion of patients with ≥ 1 claim in each period for procedural measures.

^d^ Nasal medications include classes of nasal sprays; combination sprays include two nasal medication classes.

Table S3 presents RRs after trimming 1%, 2%, and 5% of top utilization outliers to test whether observed RRs are being driven by a small fraction of extreme patients. Table S6 shows the top diagnosis codes for all E&M visits. Removal of the top 1%–5% of utilizers for E&M visits showed a fragile effect for critical care, hospital inpatient, and nursing facility visits, indicating the effect was driven by a subset of extreme outliers. The top 1% outliers accounted for 44% of post‐index critical care, 48% of hospital inpatient, and 37% of nursing facility claims. Utilization decreases observed in other E&M visit types were durable, reflecting a robust decrease not due to extreme outliers; the top 1% outliers only accounted for 8% of office/outpatient, 10% of preventative care, 12% of telemedicine, and 13% of emergency department claims (Table S3). Table S6 shows that top diagnosis codes were not NAO‐related diagnoses.

**FIGURE 1:**
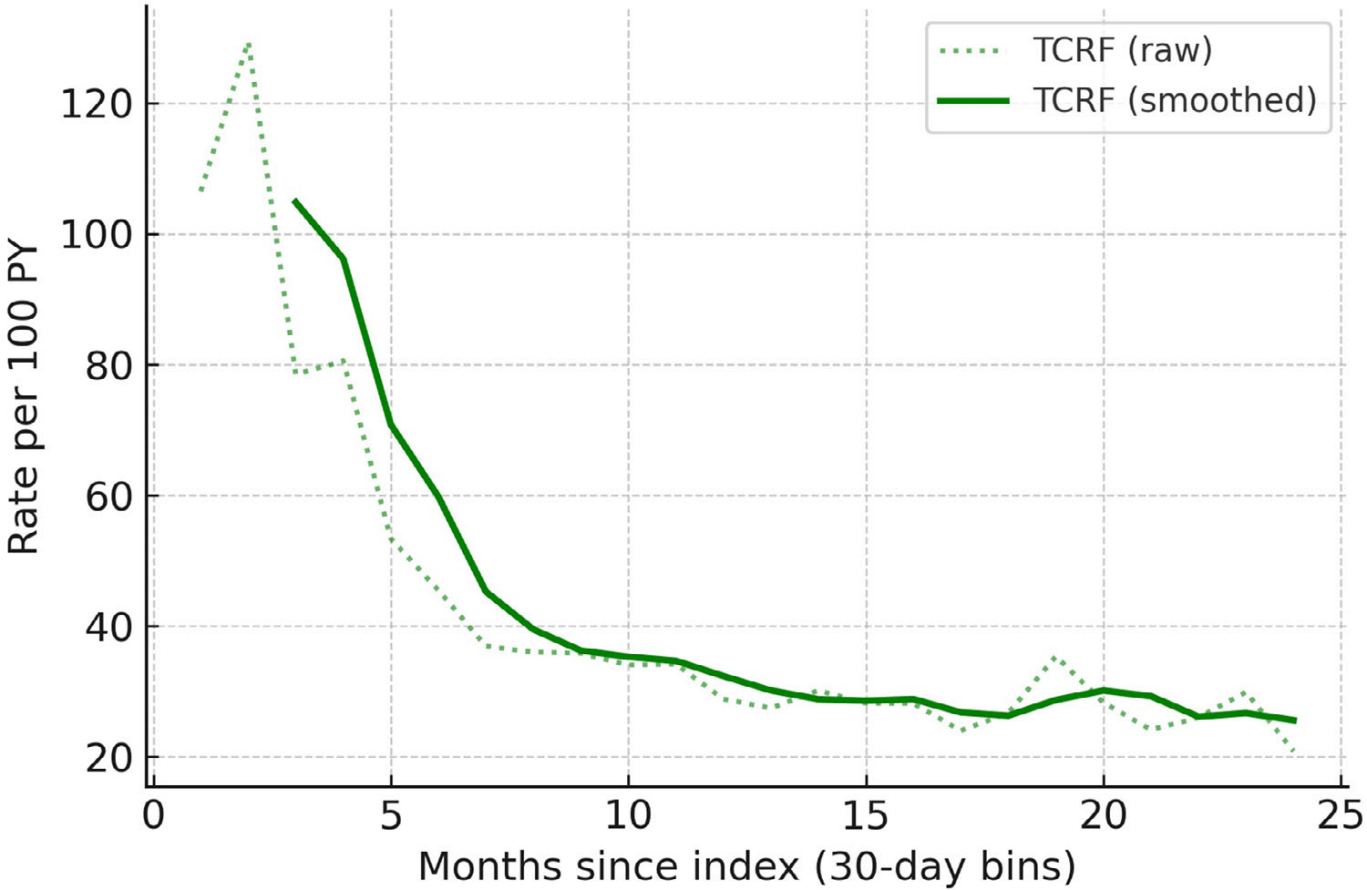
Rolling nasal‐related procedure rates in the analyzed TCRF cohort. Rates are expressed as events per 100 patient‐years, aggregated into 30‐day bins and smoothed with a rolling average.

Rhinologic procedure utilization shifted toward fewer interventions following TCRF. Balloon sinuplasty claims dropped 39.3% (135 to 82 claims), nasal endoscopy (diagnostic and therapeutic) decreased 42.9% (2189 to 1250 claims), septoplasty decreased 62.8% (273 to 101 claims), and turbinate resection/reduction decreased 64.7% (451 to 159 claims). Significant reductions were observed in all nasal‐related procedure rates utilization post‐index (Table S2). Rolling 3‐month rates/100 PY declined sharply during the first 4–6 months, stabilizing at a substantially lower level for the remainder of follow‐up (Figure 1). This sustained reduction in procedure rates suggests durable decreases in the need for subsequent nasal interventions after TCRF treatment.

**FIGURE 2:**
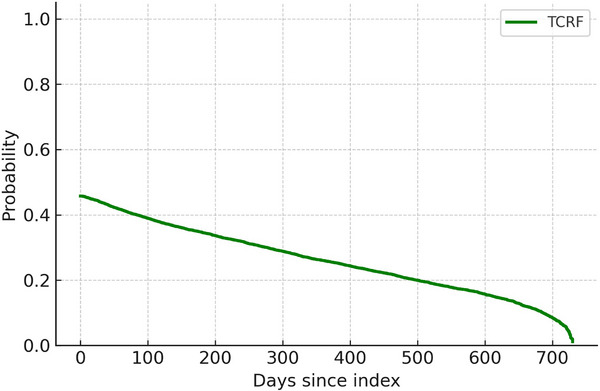
Time to last nasal‐related medication spray in the TCRF cohort. The curve shows the probability of continuing nasal‐related medication use over time following the index procedure. Patients with no post‐index fills were assigned a time of zero.

Fill rates for nasal sprays declined following TCRF but increased for MM patients in the post‐index period. In the TCRF cohort, post‐index antihistamine claims fell 26.6% (7084 to 5201 claims) and nasal corticosteroid use declined 23.9% (9564 to 7276 claims). Combination steroid–antihistamine sprays increased 31.6% (709 to 933 claims) but represented only 4.1% of the combined steroid and antihistamine nasal spray claims. In contrast, all three nasal spray categories increased significantly in the MM cohort (Table 3). Time‐to‐event analysis demonstrated a rapid decline in the probability of continuing nasal‐related medication use after TCRF treatment (Figure 2). Nearly half of TCRF patients had no post‐index nasal‐related medication fills; the Kaplan–Meier curve showed a steep early drop within the first several months, declining over the remaining 2‐year follow‐up.

### 3.3 Sleep‐Related HRU

In the pre‐index period, the TCRF cohort had a higher prevalence of sleep‐related HRU in all sleep‐related categories compared to MM patients (Table 4). Statistically significant rate reductions were observed post‐index across all sleep utilization categories in the TCRF cohort, while rates increased somewhat in the MM cohort. These findings strongly suggest that TCRF may reduce sleep‑related diagnostic and treatment burden, where RRs are < 1 with narrow CIs. Sleep‐related utilization codes, prevalence, and rates of sleep claims per CPT/HCPCS code and medication refills/generic medication are provided in Tables S1, S4, and S5.

**TABLE 4:** Sleep‐related resource utilization by category pre‐ and post‐index: TCRF versus medically managed cohort.

Category	Cohort	Prevalence^a^ (%) pre	Prevalence^a^ (%) post	Rate/100^b^ (pre)	Rate/100^b^ (post)	Rate ratio (95% CI), *p*
Sleep medications (all fills)	TCRF	9.47	5.78	22.36	13.79	0.62 (0.59–0.65), *p *< 0.001
	MM	2.48	2.37	8.94	10.11	1.13 (1.09–1.17), *p *< 0.001
Sleep study (all)	TCRF	9.17	3.93	8.46	3.26	0.39 (0.35–0.42), *p *< 0.001
	MM	4.66	4.84	5.85	6.8	1.16 (1.11–1.21), *p *< 0.001
OSA procedures (all)	TCRF	0.68	0.41	0.59	0.36	0.61 (0.45–0.81), *p *< 0.001
	MM	0.15	0.22	0.21	0.32	1.54 (1.25–1.89), *p *< 0.001

Abbreviations: MM, medically managed cohort; OSA, obstructive sleep apnea; PY, patient years; TCRF, temperature‐controlled radiofrequency cohort.

^a^ Prevalence is calculated as the proportion of patients with ≥ 1 claim in each period for procedural measures.

^b^ Rates are expressed in 100 patient‐years, adjusted for individual follow‐up time. Rate ratios 95% confidence intervals are derived from Poisson approximation with *p* values from exact two‐sample Poisson tests. A two‐sided *p* < 0.05 is considered significant.

### 3.4 Revision Rates

Among the TCRF cohort, 288 (2.82%) underwent revision by 2 years; the time of follow‐up was 434 days, and the median time to revision was 210 days (IQR 112–376). The majority of these were septoplasty (CPT 30520, 77%), followed by functional rhinoplasty (CPT 30465, 20%). No repeat TCRF procedures (CPT 30469) were identified as a first revision.

### 3.5 Cost Analysis Outcomes

**FIGURE 3:**
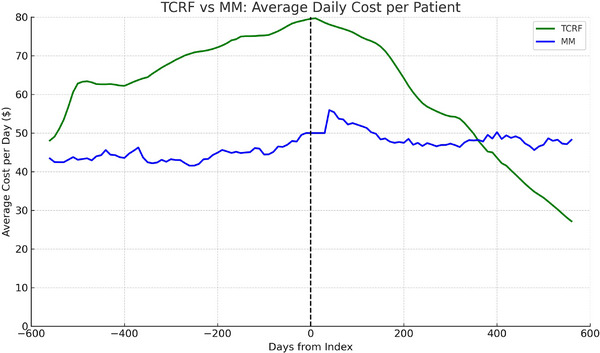
Mean daily normalized cost per covered patient over time in the TCRF and matched MM cohorts, shown relative to the index procedure. Costs are expressed in USD, normalized per covered day, and plotted over a ± 24‐month window (trimmed to ± 730 days). Smoothed curves (3‑month rolling average) are displayed for interpretability. MM, medically managed; TCRF, temperature‐controlled radiofrequency.

The TCRF cohort showed a large reduction in mean daily costs post‐index costs: $68.07 pre‐index to $38.75 post‐index (−43.1%). In contrast, mean daily costs increased in the MM cohort: $42.08 pre‐index to $63.26 post‐index (50.4%). This translates to total cost reductions of $21,418.28 in the payer‐relevant 24‐month post‐index period for TCRF and a total increased cost of $15,471.99 in the MM cohort (Table 5 and Figure 3). As shown in Figure S1, propensity weighting reduces residual baseline imbalances and clarifies separation of post‐index trajectories between cohorts, improving interpretability of weighted between‐cohort summaries, leaving the within‐patient pre/post analyses as the primary inference. Figure S2 shows a longitudinal analysis of the MM mean daily cost persists through 4 years post‐index. Table 6 shows that daily costs are consistent across data follow‐up timeframes, showing that pre‐/post‐daily costs dollars are consistently robust regardless of the amount of follow‐up time. To present payer‐relevant magnitudes, a standardized 24‐month cost as mean daily × 730.5 days was computed; thus, individuals with < 24 months of coverage contribute via their normalized daily rates rather than raw totals. Table 6 confirms that mean daily costs are consistent across follow‐up durations.

**TABLE 5:** Daily, monthly, 24‐month in costs pre‐ and post‐index.

Cost type	TCRF cohort (*N *= 10,206)	MM cohort (*N *= 50,766)
Mean daily cost^a^, pre‐index procedure	$68.07 95% CI $66.36–$69.78	$42.08 95% CI $40.79–$43.79
Mean daily cost^a^, post‐index procedure	$38.75 95% CI $37.53–$39.97	$63.26 95% CI $54.44–$89.94
Mean change in daily cost^a^ pre‐ and post‐index procedure	−$29.32 95% CI $30.87–$27.78	$25.60 95% CI $16.78–$52.39
Mean monthly^b^ cost, pre‐index procedure	$2071.88 95% CI $2019.83–$2123.93	$1,280.81 95% CI $1241.55–$1332.86
Mean monthly^b^ cost, post‐index procedure	$1179.45 95% CI $1142.32–$1216.59	$1925.48 95% CI $1657.02–$2737.55
Total 24 month^c^ cost pre‐index procedure	$49,725.14 95% CI $48,475.98–$50,974.29	$30,739.44 95% CI $29,797.10–$31,988.60
Total 24 month^c^ cost post‐index procedure	$28,306.88 95% CI $27,415.67–$29,198.09	$46,211.43 95% CI $39,768.42–$65,701.17
Mean index cost^d^	$4938.19 95% CI $4710.46–$5165.92	$610.54 95% CI $591.42–$631.62

^a^ Mean daily cost is normalized per covered day, not exceeding ± 24 months index.

^b^ Average calendar month (365.25 days   12 months).

^c^ 24 Calendar months (730.5 days).

^d^ Mean index cost $4492.19 after removing top and bottom 1% outliers; median index cost $4374.50.

**TABLE 6:** Sensitivity analysis of daily costs by minimum post‐index follow‐up time requirement: TCRF cohort.

Subset	*N*	Pre‐index mean daily cost (95%CI)	Post‐index mean daily cost (95%CI)
All follow‐up	10,206	$68.07 (66.36, 69.78)	$38.75 (37.53, 39.97)
≥ 3 months	9345	$69.08 (67.27, 70.89)	$37.86 (36.64, 39.08)
> 6 months	8152	$68.37 (66.46, 70.29)	$36.85 (35.61, 38.10)
≥ 12 months	5841	$68.70 (66.45, 70.94)	$36.43 (34.98, 37.87)
≥ 18 months	3775	$67.06 (64.30, 69.81)	$35.27 (33.52, 37.01)
≥ 24 months	1487	$65.85 (61.47, 70.24)	$33.67 (31.07, 36.28)

## 4 Discussion

TCRF is a minimally invasive procedure to restore nasal patency with consistent clinical outcomes across multiple studies, demonstrating > 90% responder rates of significant symptom improvement within a month of treatment, sustained through follow‐up periods out to 4 years [11, 17, 18, 19]. This is where most evaluations of promising procedures conclude. Building on established evidence supporting the safety and efficacy of TCRF for NVC‐related NAO, the present study leveraged RWD from thousands of patients to demonstrate its practical health and economic benefits in routine clinical practice.

The MM cohort's index event (initial NAO diagnosis/medical therapy) anchors patients early in their care trajectory, and utilization rises thereafter as evaluation and medical management are optimized. In contrast, the TCRF cohort index event is procedurally selected with higher observed pre‑index NAO‑related burden, consistent with the structural nature of NVC‐related NAO and limited responsiveness to conservative therapy. TCRF yielded meaningful exposure‑adjusted reductions across visits, procedures, and nasal sprays. Persistent post‑TCRF levels slightly above post‑MM in medication classes (e.g., corticosteroid sprays) likely reflect coexisting allergic/inflammatory disease not directly targeted by nasal valve remodeling.

The ideal profile for medical technology in a cost‐constrained healthcare system is one that simultaneously improves care quality while decreasing total care costs. Significantly, the total cost‐of‐care savings realized by TCRF treatment of NVC‐related conditions are driven by NAO‐related utilization decreases across the board—spanning in‐office visits to primary care physicians and specialists, ENT‐related procedures, NAO medications, and sleep‐related care.

The findings of this study confirm the importance of NAO in relation to overall health, including sleep. Nasal obstruction and sleep disturbance have previously been related to daytime somnolence, presenteeism at work, and depression [4, 7, 8, 9]. The broad impact of NAO and NVC on overall health would appear to be confirmed with this RW analysis of health care claims following intervention. The nasal valve is the narrowest part of the nasal airway, and even minor increases in airway diameter in this critical area can have profound effects (e.g., by conceptually applying Poiseuille's Law, increasing the radius of the nasal airway from 1 to 2 mm results in a potential 16‐fold increase in nasal airflow).

In stark contrast to the matched MM NAO patients, which had mean daily costs increasing by 50.4% over the post‐index period, the TCRF group saw a substantial 43% cost savings in these same costs in the same period. The cohort of MM patients also had upward trends in multiple types of HRU during the post‐index period—increases not seen in the TCRF‐treated patients. This increases confidence in the results of the TCRF patients, that the decreases in NAO‐related HRU and total cost‐of‐care were not spurious. Considering that there are often multiple and sometimes overlapping causes of NAO and NVC‐related NAO is primarily a structural disease, these findings suggest that addressing the anatomic cause is an effective method for addressing NVC‐related NAO as a structural disease. While medical management may address inflammatory causes of NAO, it doesn't address structural causes, especially NVC.

Inherent in any evaluation of minimally invasive procedures is the evaluation of revision rates—including the need for escalation to more extensive surgeries. This research demonstrated a low RW revision rate of 2.8% across 10,206 patients with a mean time of follow‐up of 434 days and a median time to revision of 210 days, consistent with controlled study results documenting durable symptom improvement and low reintervention rate of 3% [17, 18, 19].

Strengths of this study include the use of a large, nationally representative, RW claims database, precise case identification via a dedicated CPT code (30469), comprehensive capture of costs, and inclusion of a matched cohort reflecting MM patients with NAO. The breadth of the dataset enhances the generalizability of findings across routine practice, spanning coverage types, geographic regions, and patient subgroups.

This study has several limitations to be considered when interpreting the results. First, the analysis relied exclusively on CPT code 30469 to identify TCRF procedures. As such, it may have excluded TCRF procedures predating code introduction or mis‐coded. Second, we excluded cases involving concurrent sinonasal procedures such as septoplasty or sinus surgery within the index period. This decision was made to isolate the economic impact of TCRF alone, but it doesn't fully reflect clinical practice that includes multimodal surgical management. Third, claims data don't include over‐the‐counter medication use, indirect costs, and patient‐reported outcomes, meaning the full impact of TCRF procedures may be underestimated. As a mitigation, we introduced several controls, including a pre‐index period for cost‐normalization, daily tracking of attrition, and enforcement of coverage requirements to address incomplete data capture. Finally, because this analysis used US administrative claims with US coding and payer structures, external validity outside the United States may be limited, as comparative costs and care pathways may differ in other health systems. The same RWD framework could be applied to evaluate other NAO interventions to inform setting‑specific value assessments.

Despite these potential limitations, the analysis delivered on our objectives to study all patients with isolated TCRF for NVC‐related NAO, tracking utilization and costs across a continuous enrollment period before/after the procedure and comparing daily costs over time to MM patients. The fact that these patients, especially those of Medicare age with their attendant co‐morbidities, would still pursue a procedure for their NVC‐related NAO suggests a significant disease burden.

## 5 Conclusion

This is the first study to demonstrate that TCRF is associated with significant HRU and cost reductions across multiple categories of care: E&M visits (predominantly primary care and ENT office visits), procedures (septoplasty, FESS, balloon sinuplasty, nasal endoscopies, and rhinoplasty), nasal medications (steroid, antihistamine, anticholinergic, and decongestant sprays), and sleep (tests, medications, and procedures). The drivers of the cost savings were NAO‐related in each category, resulting in significant decreases in total cost of care ($68.07 daily costs pre‐ to $38.75 post‐index period [−43%]), in contrast to the costs incurred by the MM cohort ($42.08 pre‐ to $63.26 post‐index period [+50%]). The study highlights the importance of NAO in overall patient health.

## Funding

This study was funded by Aerin Medical.

## Conflicts of Interest

David W. Kennedy: Consultant for Kenvue, Glaxo Smith Kline, Sanofi, Regeneron, Aerin Medical, Royalties Medtronic. Gavin Setzen: Consultant for Stryker ENT, Smith + Nephew, Polyganics Inc., Aerin Medical. Ashleigh A. Halderman: Consultant for Aerin Medical. Kevin C. Welch: Advisor for GlaxoSmithKline, Consultant for Aerin Medical, Board of Directors for American Rhinologic Society. Bobby Tajudeen: No Disclosures. Gary M. Owens: Consultant for Aerin Medical, Baxter, Sanofi, Regeneron, Vedanta Biosciences, Rocket Pharmaceuticals. Paul J. Niklewski: Consultant for Aerin Medical. Masayoshi Takashima: Consultant for Aerin Medical, Medtronic, Spirair.

## Supporting information




**Supporting Table 1**: Sleep‐related Utilization Codes: TCRF and Medically Managed Cohorts.
**Supporting Table 2**: ENT Procedure Utilization by CPT Code Pre‐ and Post‐Index TCRF.
**Supporting Table 3**: Fragility and Durability Trimming Results for Evaluation and Management Visit Categories: TCRF Cohort.
**Supporting Table 4**: Sleep‐related Healthcare Resource Utilization by CPT/HCPCS Codes: TCRF Cohort.
**Supporting Table 5**: Sleep Medications (all Fills/Refills): TCRF cohort.
**Supporting Table 6**: Top 5 Diagnoses Overall vs Top 1% Utilizers.
**Supporting Table 7**: Baseline characteristics of the TCRF and MM cohorts before and after ATT weighting: TCRF Cohort.
**Supporting Figure 1**: Cost trends in TCRF and MM cohorts, comparing weighted and unweighted estimates. (95% CI; monthly dashed, smoothed solid).
**Supporting Figure 2**: Average patient daily cost comparison of TCRF versus MM, extending MM out to 48 months post‐index.
**Supporting Figure 3**: Fragility and durability trimming curves for E&M utilization categories. Panels display rate ratios across trimming thresholds with 95% confidence intervals.
**Supporting Figure 4**: Robustness checks using winsorization (95th–99th percentile caps) and leave‐k%‐out resampling. Curves illustrate stability of key utilization findings under these sensitivity analyses. (blue = fragility, green = durability).
