# Supplementary material for: Healthcare Resource Utilization and Cost After Temperature‐Controlled Radiofrequency Treatment of Nasal Airway Obstruction: A Real‐World Longitudinal Claims Analysis
Source: Int Forum Allergy Rhinol. 2025 Nov 26;16(3):261–71. doi: 10.1002/alr.70066 (PMC12951823; doi:10.1002/alr.70066)
Supplement: Supplementary file 1 — Supporting Table 1: Sleep‐related Utilization Codes: TCRF and Medically Managed Cohorts. Supporting Table 2: ENT Procedure Utilization by CPT Code Pre‐ and Post‐Index TCRF. Supporting Table 3: Fragility and Durability Trimming Results for Evaluation and Management Visit Categories: TCRF Cohort. Supporting Table 4: Sleep‐related Healthcare Resource Utilization by CPT/HCPCS Codes: TCRF Cohort. Supporting Table 5: Sleep Medications (all Fills/Refills): TCRF cohort. Supporting Table 6: Top 5 Diagnoses Overall vs Top 1% Utilizers. Supporting Table 7: Baseline characteristics of the TCRF and MM cohorts before and after ATT weighting: TCRF Cohort. Supporting Figure 1: Cost trends in TCRF and MM cohorts, comparing weighted and unweighted estimates. (95% CI; monthly dashed, smoothed solid). Supporting Figure 2: Average patient daily cost comparison of TCRF versus MM, extending MM out to 48 months post‐index. Supporting Figure 3: Fragility and durability trimming curves for E&M utilization categories. Panels display rate ratios across trimming thresholds with 95% confidence intervals. Supporting Figure 4: Robustness checks using winsorization (95th–99th percentile caps) and leave‐k%‐out resampling. Curves illustrate stability of key utilization findings under these sensitivity analyses. (blue = fragility, green = durability). [file ALR-16-261-s001.docx]

**Appendix 1: Supplemental Methods**

### **Data Source and Cohort Construction**

The analysis used de‑identified, patient‑level claims from the **Komodo Healthcare Map™ (Sentinel)** environment hosted in **Snowflake**, with data available from **January 2016 through June 2025. T**emperature‑controlled radiofrequency (TCRF) cases occurred after the introduction of a dedicated CPT code in 2023. Analyses incorporated **inpatient, outpatient, and pharmacy** claims and associated estimated **allowed amounts** to compute utilization and cost outcomes.

Three cohorts were constructed. First, a **general NAO population** was identified as patients with ≥1 claim bearing an ICD‑10‑CM diagnosis for nasal airway obstruction (NAO): **J34.2, J34.3, J34.82, J34.89, J34.9, J34.8200–J34.829, and M95.0**; **R09.81** (nasal congestion) was reserved for sensitivity analyses. Notably, **nasal valve collapse–specific codes (J34.8200–J34.829)** were introduced on **October 1, 2024** and were applied prospectively from that date, with earlier NAO diagnoses captured using pre‑existing codes.

Second, an **isolated TCRF cohort** was defined using **CPT 30469** (nasal valve repair, TCRF). This code was established January 1, 2023. The **index date** for each patient was the earliest 30469 service date within the index episode. Where multiple 30469 codes existed for a patient, codes occurring within two **days** were treated as a single episode to avoid duplicating the index. To isolate the effect of TCRF, patients were **excluded** if any predefined **nasal/sinonasal procedures** occurred **within ±2 days** of the index date. The exclusion list comprised **31242, 31295, 31296, 31297, 31298, 30520, 30140, 42975, 00160, 30130, 31267, 61782, 31276, 31256, 31240, 31254, 42826, 69706**, capturing balloon dilations, septoplasty/turbinates, endoscopic sinus procedures, adjuncts (e.g., stereotactic navigation), anesthesia, and other nasal interventions that could confound attribution of post‑index outcomes to TCRF. A **±2‑day peri‑index window** around the index was excluded from utilization summaries to avoid misattributing episode‑of‑care services to baseline or follow‑up.

Third, a **matched NAO Medically Managed (MM) cohort** of medically managed patients was drawn from the NAO population, requiring **no sinonasal procedures** from the exclusion list during the pre‑ and post‑index observation windows, and was assigned an index date as the first NAO diagnosis. Matching enforced **sex** concordance (with unknown sex permitted to match either category), **age within ±5 years**, **pre‑index total cost within ±25%**, and **Charlson comorbidity index within ±25%**. A **maximum‑cardinality bipartite matching** algorithm was then applied so that each TCRF patient was matched to **≥5 unique patients**, with each MM patient used at most once, maximizing sample size under the caliper constraints; between‑cohort descriptive summaries for the MM group were presented using **Average Treatment effect on the Treated (ATT) weights**, with unweighted analyses used for sensitivity checks.

Coverage (closed‑claims) windows from enrollment files defined **pre‑index** (up to **−730 to −3** days), **index** (**−2 to +2** days), and **post‑index** (**+3 to +730** days) periods; a minimum of 3 **months** of continuous pre‑index coverage and up to **24 months** of post‑index coverage were required where available. Denominators for rates and daily cost normalization were limited to **days with coverage**, with days without coverage excluded to prevent denominator bias. Costs were summarized as **total** allowed amounts within each window and **normalized per covered day**, with linear transformations to monthly and annual values used for interpretability in figures and tables.

Operationally, cohort construction and claim assembly were executed in **Snowflake** using common‑table expressions to: (i) identify valid 30469 index episodes and dates; (ii) apply the **±2‑day** concomitant‑procedure exclusions using the code list above; (iii) join coverage and demographics; and (iv) union **non‑inpatient** (CPT/HCPCS), **inpatient** (DRG with arrays of CPT/HCPCS and revenue codes), and **pharmacy** (NDC) claim sources into a unified patient‑day panel keyed by **DAYS_FROM_INDEX**. Pharmacy NDCs were mapped to **precise generic names** via **SENTINEL_COMMON.SENTINEL_REFERENCE.DRUG_REFERENCE** to support class‑level medication analyses. Estimated allowed amounts were retained from all claim sources to compute pre/index/post **total** and **daily normalized** costs in the TCRF and MM cohorts.

**MM Design Justification**

The choice to **coarsen age** to **±5 years** followed established practice of **coarsened exact matching** (CEM), which improves overlap and reduces model dependence by enforcing balance on clinically salient variables ex ante.^1^ Direct **calipers on strong prognostic covariates**—specifically **pre‑index total cost** and **Charlson index**—were set at **±25%** to enforce near‑neighbor matching on baseline resource use and illness burden while preserving adequate match availability; this approach is consistent with the general caliper‑matching literature in which narrow calipers on highly prognostic distances reduce residual bias. ^2^ Allowing a **variable number of MMs per treated subject** and optimizing **match cardinality** increases precision and can yield larger bias reduction than fixed 1:1 schemes, particularly in large claims datasets.^3^ Modern **cardinality matching** methods provide a principled way to maximize the number of matched MMs subject to prespecified balance constraints and have been recommended for designing balanced, representative samples in observational studies.^4^

The use of **ATT‑weighted** MM summaries after matching aligns the MM cohort to the treated cohort’s baseline profile and is consistent with best practices for matched/weighted analyses in causal inference.^5^ The **±2‑day peri‑index exclusion** around the operative date is standard in episode‑of‑care construction to prevent misattribution of services temporally tied to the index procedure. Finally, the **Charlson comorbidity index** was computed from ICD‑10‑CM using the **Quan** coding algorithms, which have been widely validated for administrative datasets and are cited in the manuscript.^6^

### **Charlson Comorbidity Assessment**

Comorbidity burden was assessed using the Charlson comorbidity index derived from inpatient and outpatient claims in the 12-month pre-index window. ICD-10-CM diagnoses were mapped to Charlson categories using the Quan adaptation, which has been validated for use in administrative datasets.^7^ Diagnosis codes were normalized to uppercase alphanumeric strings with punctuation removed so that variations in formatting (e.g., “J34.2,” “J342,” “j34.20”) were treated equivalently. Built-in hierarchies were respected to prevent double counting; for example, “diabetes with complications” superseded “uncomplicated diabetes.”

Two forms of the Charlson index were generated for each patient. First, an **unweighted comorbidity count** represented the number of Charlson categories present. Second, a **weighted Charlson score** was calculated as the sum of category indicators multiplied by the published Quan weights (e.g., myocardial infarction = 1, renal disease = 2, metastatic tumor = 6). Both metrics were calculated at the patient level for TCRF and MM cohorts.

For comparability, the same Charlson variables were constructed in both the TCRF and matched MM cohorts. These variables were used both for baseline characterization of disease burden and as part of the matching calipers, where MMs were required to fall within ±25% of the treated patient’s Charlson weighted index to be eligible. Cohort balance in Charlson scores and weighted scores was assessed before and after ATT weighting.

This approach ensured that the matched MM cohort carried a comorbidity profile comparable to the treated cohort, thereby minimizing confounding by baseline disease burden while preserving sufficient sample size for analysis.

**ATT Weight Development**

Average treatment effect on the treated (ATT) weights were developed to align the matched MM cohort to the treated TCRF cohort on baseline characteristics while accounting for severity. Pre-index data tables contained patient-level summaries of costs, claims, and diagnosis proxies, drawn separately for TCRF and MM patients. Diagnostic proxies included nasal airway obstruction–related codes (septal deviation, turbinate hypertrophy, nasal valve collapse, allergic rhinitis, and chronic rhinosinusitis), the number of distinct NAO diagnosis days, and binary indicators for presence of each condition.

The ATT weighting proceeded in several steps. First, each patient was assigned to a severity stratum defined by pre-index ear, nose, and throat (ENT) utilization and nasal spray intensity. Strata indicators incorporated whether any ENT or nasal spray claims were present, the frequency of ENT encounters, and the intensity of nasal spray use, resulting in discrete strata that separated low-utilization from higher-intensity patients. Within each stratum, a set of continuous log-transformed covariates was constructed, including log costs and claim counts for ENT services, sprays, pharmacy, and total medical spending, as well as the number of claim days. Diagnosis proxies, age, and sex were also included.

Within each severity stratum, a logistic regression propensity score was estimated predicting TCRF treatment as a function of the available covariates. Propensity scores were constrained between 0.001 and 0.999 to avoid extreme values. ATT weights were then derived such that treated patients received a weight of 1.0, and MM patients received a weight equal to p/(1–p), where p was the estimated propensity of treatment. To avoid instability due to extreme weights, the upper and lower 1% of MM weights within each stratum were trimmed to the 1st and 99th percentiles.

Weighted balance diagnostics were then produced for each stratum. Standardized mean differences (SMDs) were calculated for each covariate before and after weighting, both within strata and in aggregate. Results were exported as patient-level weights and a balance table of covariate SMDs. Console checks and tabular outputs confirmed that post-weighting balance improved substantially across key utilization and diagnosis proxies, with most weighted SMDs approaching zero, indicating adequate alignment between treated and weighted MM cohorts.

This approach extended traditional ATT weighting by layering severity-based stratification (ENT × spray use × intensity) prior to estimation of propensity scores. By combining stratification and weighting, the method preserved clinical comparability across levels of baseline severity while reducing imbalance in continuous utilization and cost measures. Weighted results were then used throughout between-cohort summaries to ensure the MM group reflected the treated cohort’s baseline profile, with unweighted results retained for sensitivity analyses.

**ATT Weighting Rationale**

The ATT weighting strategy was chosen to ensure that the MM cohort not only matched the treated cohort on broad demographic and comorbidity factors, but also reflected differences in baseline severity of NAO presentation. Simple 1:1 matching can reduce imbalance but often discards large amounts of data, and even conventional ATT weighting can struggle when utilization is highly skewed. By first stratifying patients on pre-index ENT and nasal spray intensity, heterogeneity in disease management severity was preserved, and balance was improved within clinically interpretable subgroups. This stratified approach follows the logic of subclassification in observational studies, which has long been recommended to reduce residual bias before modeling propensity scores. ^8^ Within each stratum, propensity scores were estimated using a logistic model, and ATT weights were applied to align MMs to the treated distribution. Trimming extreme weights and capping at the 1st and 99th percentiles is a standard practice to mitigate the influence of outliers, thereby improving stability and effective sample size.^9^

The addition of balance diagnostics ensured that the weighting achieved its intended purpose. Standardized mean differences (SMDs) before and after weighting are widely recognized as the most informative measure of covariate balance, with values <0.1 typically considered acceptable (Austin PC. Balance diagnostics for comparing the distribution of baseline covariates between treatment groups in propensity-score matched samples.^10^ The computation of effective sample size provided a practical assessment of precision loss due to weighting and allowed confirmation that the weighted MM sample retained sufficient information for inference.

By incorporating severity stratification, trimming of extreme weights, and comprehensive balance diagnostics, the ATT approach used in this study reflects best practices in observational data analysis. This ensured that subsequent comparisons of TCRF patients and MMs represented differences attributable to treatment rather than artifacts of baseline severity or cost heterogeneity.

In the weighting/matching design, **NAO/NVC disease‑state indicators and ENT‑specific utilization** (pre‑index ENT costs/claims, nasal spray intensity, NAO diagnosis frequency) was deliberately prioritized over global comorbidity indices. This choice reflects the estimand, **the average treatment effect in patients presenting with clinically relevant NAO/NVC severity**, and is consistent with evidence that **disease‑specific pre‑index utilization is more prognostic of post‑index ENT utilization/costs** than aggregate comorbidity burden. Consequently, **Charlson index variables were retained (calipered at ±25%) to guard against major imbalance** but were **ranked lower** than ENT severity in the propensity/weighting steps. Charlson is reported transparently in baseline tables and assess residual balance after ATT weighting. Where modest residual imbalance remains, **sensitivity analyses** re‑estimating weights with Charlson promoted to a higher‑priority balance constraint yield the same qualitative conclusions.

### **Utilization Analyses**

Nasal airway–related utilization was measured across procedures, medications, and evaluation and management (E&M) encounters. Procedures were defined using CPT and HCPCS codes listed in Supplemental Table S1, including septoplasty, turbinate surgery, balloon dilation, functional endoscopic sinus surgery components, and other nasal interventions. Pharmacy utilization was mapped from claims at the generic-drug level and grouped by therapeutic class, with categories including antihistamines, leukotriene modifiers, intranasal corticosteroid sprays, anticholinergics, decongestants, and combination sprays. Encounters billed under E&M codes were further classified into outpatient/office visits, hospital inpatient and observation stays, emergency department visits, preventive services, telemedicine and remote encounters, nursing facility visits, and critical care encounters.

Sleep-related utilization was assessed separately, reflecting the clinical link between nasal obstruction and sleep disturbance. Diagnostic testing included polysomnography (CPT 95810 and 95811) and home sleep apnea testing (HCPCS G0398–G0400). Procedures included pharyngoplasty for obstructive sleep apnea (OSA; CPT 42975). Sleep-related pharmacotherapy was captured at the generic level, as detailed in Supplemental Table S5, and encompassed benzodiazepines, non-benzodiazepine hypnotics, melatonin receptor agonists, orexin antagonists, and selected antidepressants with sleep indications.

For all utilization endpoints, prevalence was defined as the proportion of patients with at least one claim in the pre- or post-index period. Event rates were standardized for unequal follow-up by expressing outcomes per 100 patient-years (PY) of observed coverage. This metric was chosen to account for variation in closed-claims coverage windows and provides a denominator that is interpretable across diverse claim types. Rate ratios with 95% confidence intervals were derived from Poisson models, and paired analyses were performed within the TCRF cohort to compare pre- and post-index periods.

### **Time-to-Event Analyses**

Two survival-type analyses were conducted to examine patterns of treatment discontinuation and procedure utilization over time.

For nasal-related pharmacotherapy, the **event** was defined as the final post-index prescription fill for any medication in the nasal airway therapeutic categories (antihistamines, leukotriene modifiers, intranasal corticosteroids, anticholinergics, decongestants, or combination sprays). Patients who had no post-index fills were assigned an event time of zero days. Patients whose last fill occurred within 60 days of the end of their coverage window were censored, reflecting the possibility of ongoing use at the time of disenrollment. Time from the index procedure to medication cessation was analyzed using a Kaplan–Meier framework, with discontinuation probability shown over 24 months of follow-up.

For nasal-related procedures, monthly utilization rates were computed over the post-index period. Claims were aggregated into 30-day bins, and the number of procedure claims in each bin was standardized by person-time at risk, expressed as events per 100 patient-years. To reduce short-term volatility and highlight broader patterns, a **three-month rolling average** was applied to the monthly rates. These smoothed trajectories were plotted to illustrate the temporal decline in procedure utilization after TCRF treatment.

This dual approach provided complementary perspectives: the Kaplan–Meier curves characterized the rapid discontinuation of nasal medications at the patient level, while the rolling rate analysis depicted population-level changes in procedural demand over time.

### **Revision Procedures**

Revision procedures were identified in the 24-month post-index period using CPT codes associated with repeat treatment of the nasal valve or escalation to more extensive surgical interventions. Revisions included **repeat TCRF nasal valve repair (CPT 30469)**, **functional rhinoplasty procedures involving alar or nasal valve repair (CPT 30465, 30420, 30400)**, and the **placement of a bioabsorbable nasal implant (Latera®, CPT 30468)**.

Each patient’s time to revision was calculated as the number of days from the index TCRF procedure to the first occurrence of any of the revision procedure codes. Patients without a revision were censored at the end of their available follow-up. Revision rates were reported both as the proportion of patients undergoing any revision and stratified by procedure type. This framework allowed characterization of both the **overall durability of TCRF** and the **type and timing of secondary interventions** required in routine practice.

### **Cost Analyses**

Cost outcomes were derived from allowed amounts across inpatient, outpatient, and pharmacy claims. Costs were assigned to three non-overlapping windows relative to the index date: the pre-index window (−730 to −3 days), the index window (−2 to +2 days), and the post-index window (+3 to +730 days).

For each patient, total costs within each window were calculated and normalized by the number of covered days in that window. This produced daily costs that account for differences in enrollment duration. Daily costs were then converted to monthly and annualized values by linear transformation (e.g., daily cost multiplied by 30.44 for monthly and by 730.5 for two-year projections). Days with coverage but no claims contributed zero cost, while days without coverage were excluded. Not all patients had 24 months of coverage and values are scaled linear transformations based on the normalized cost.

MM cohort summaries were weighted using average treatment effect on the treated (ATT) weights to reflect the treated cohort’s baseline distribution. Unweighted analyses were also performed as sensitivity checks. Bootstrapped confidence intervals were estimated from patient-level resampling to account for skewed cost distributions. Paired pre/post comparisons within the TCRF cohort were conducted using Wilcoxon signed-rank tests, while between-cohort comparisons relied on weighted means and corresponding confidence intervals.

This approach provided estimates of total and normalized costs before and after TCRF, allowed comparison with medically managed MMs, and enabled calculation of the change in daily healthcare cost attributable to treatment. Sensitivity analyses evaluated whether findings were robust to weighting method, alternative denominators, and trimming of extreme utilizers.

### **Statistical Analyses**

The primary analysis was a within-patient pre/post comparison around the index TCRF procedure. Utilization outcomes, including outpatient visits, ENT procedures, and prescription fills, were standardized for unequal follow-up by converting events to exposure-adjusted rates expressed per 100 patient-years. Pre/post differences in any-use proportions were tested with McNemar’s test, and pre/post differences in event rates were estimated using Poisson regression models to derive rate ratios with 95% confidence intervals.

Cost outcomes were analyzed using paired Wilcoxon signed-rank tests to compare pre- and post-index daily normalized costs. Between-cohort analyses applied ATT weights to align the MM cohort to the treated population. Unless otherwise specified, all MM results were reported as weighted means and rates. To quantify variability in skewed distributions, 95% confidence intervals were generated using patient-level bootstrap resampling.

Sensitivity analyses included several complementary approaches. Weighted results were compared to unweighted MM summaries to confirm consistency. Fragility and durability analyses were conducted for utilization outcomes by trimming the top 1–5% of utilizers; outcomes that attenuated toward a null effect were classified as fragile, whereas outcomes that remained stable after trimming were classified as durable. Winsorization at the 95th to 99th percentiles and leave-k%-out resampling (random exclusion of 1–5% of patients with 200 repetitions) were performed to evaluate robustness to extreme values and sample variability. Cost outcomes were tested across weighting methods and denominators to confirm stability of observed savings.

**Bootstrap resampling & confidence intervals**
Uncertainty via **patient‑level nonparametric bootstrap** with **B = 2,000** replicates was estimated unless otherwise noted. Each bootstrap replicate sampled **TCRF patients with replacement** from the analytic cohort and, for between‑cohort summaries, **sampled MM patients with replacement** from the matched pool. **Weights in the main bootstrap were held fixed** (design‑consistent bootstrap) to reflect uncertainty in outcomes conditional on the designed comparison; a **design‑stage bootstrap** that **re‑estimated ATT propensity models/weights within each replicate** (B = 200) was conducted as a sensitivity analysis and produced materially similar CIs.

For **pre/post within‑patient outcomes** (rates, any‑use, daily costs), resampling occurred at the **patient level** so that each patient’s pre‑ and post‑windows moved together. For **rate outcomes**, events were recomputed, person‑time denominators (covered days), and **Poisson RRs** in each replicate using the original model specification. For **cost outcomes**, daily normalized costs per replicate were recomputed; **95% CIs** use the **percentile method**. (Results were unchanged with BCa CIs.)

**Skew/outliers:** To assess sensitivity to heavy tails, repeated bootstraps after **winsorizing costs** (95th/97.5th/99th percentile caps) and via **leave‑k%‑out** (randomly excluding 1–5% of patients; 200 iterations). Conclusions were unchanged (see Supplemental Figure S1).

All analyses were performed in R version 4.5.1 using standard packages including dplyr, survival, comorbidity, and ggplot2.

## **Results – Sensitivity Analyses**

**Fragility and Durability of Utilization Outcomes**

Sensitivity analyses were conducted to test whether observed changes in utilization were driven by a small subset of patients or reflected broad-based effects. For categories with rate ratios greater than 1.0 at baseline, including critical care, inpatient/observation, and nursing facility encounters, patients with the highest number of post-index events were trimmed incrementally (1%, 2%, and 5%). These increases proved fragile: as shown in Supplemental Table S3 and Figure S3, rate ratios collapsed toward 1.0 when as little as 2–7% of high utilizers were removed, indicating that the apparent increases were largely explained by outliers. In contrast, for categories with rate ratios less than 1.0, including emergency department visits, office/outpatient encounters, preventive care, and telemedicine visits, trimming was applied to patients with the greatest increases in utilization. These decreases were durable: rate ratios remained below 1.0 with narrow confidence intervals after trimming, as demonstrated in Table S3 and Figure S3. Winsorization at the 95th, 97.5th, and 99th percentiles and leave-k%-out resampling with random exclusion of 1–5% of patients (200 iterations each) produced consistent results, confirming that observed reductions were not dependent on a handful of extreme patients (Figure S4).

**Weighted Versus Unweighted Cohort Comparisons**

Because MM results were presented using ATT weights to reflect the treated cohort’s baseline profile, unweighted analyses were conducted as a sensitivity check. Weighted and unweighted MM results yielded the same overall interpretation: TCRF patients demonstrated marked reductions in utilization and costs, while MMs showed stable or slightly increasing patterns over the same period. Weighted MM analyses provided closer alignment in age, sex, Charlson comorbidity, and baseline cost distributions (see Supplemental Table S7), but the concordance between weighted and unweighted estimates confirmed that the findings were robust to the choice of weighting strategy. Key comparisons for E&M utilization are summarized in Table 3 of the main manuscript and detailed in Supplemental Table S3.

**Cost Robustness Analyses**

Post-index reductions in both total and normalized daily costs were tested under multiple specifications. As shown in Supplemental Table S4 and Figure S2, reductions in daily cost remained significant across analyses conducted with and without ATT weights, with alternative denominators (per patient versus per covered day), and after excluding the top 1–5% of cost utilizers. Bootstrap resampling confirmed that confidence intervals were stable despite skewness in the cost distribution. Taken together, these findings support the conclusion that observed savings were not an artifact of weighting, denominator choice, or a small set of extreme cost cases.

**Durability/Trimming Method**

For each E&M category, observed pre/post changes were assessed to determine whether they were concentrated in a small subset of high utilizers. Let y_i,pre_ and y_i,post_ denote a patient’s pre‑ and post‑index event counts. Let PT_i,pre_ and PT_i,post_ represent covered person‑time. The baseline rate ratio (RR) was computed as:

$$RR=\frac{\sum_{i} y_{i,post}/\sum_{i} {PT}_{i,post}}{\sum_{i} y_{i,pre}/\sum_{i} {PT}_{i,ppre}}$$

$$Trimming rules:$$

- If RR>1: patients were ranked by post‑index counts y_i,post_ and the top p% (p = 1, 2, 5) were removed from both periods (removing their y_i,pre_, y_i,post_, PT_i,pre_, PT_i,post_), and RR was recomputed.
- If RR<1: patients were ranked by the increase (y_i,post_ − y_i,pre_) and the top p% (p = 1, 2, 5) increasers were removed (removing their pre/post contributions), and RR was recomputed.

A category was classified as fragile if RR crossed 1.0 at or before 5% trimming (with 95% CIs including 1.0), and durable if RR remained on the same side of 1.0 at 5%. Trimming curves with 95% CIs (Poisson approximation) were plotted, and a summary table reported the minimum trim % at which RR approached 1.0. Critical care, inpatient/observation, and nursing facility increases were fragile, whereas ED, office/outpatient, preventive, and telemedicine decreases were durable.

**References**1. (Iacus SM, King G, Porro G. Causal Inference Without Balance Checking: Coarsened Exact Matching. Political Analysis. 2012;20(1):1–24. doi:10.1093/pan/mpr013).

2. (Austin PC. Optimal Caliper Widths for Propensity‑Score Matching when Estimating Differences in Means and Differences in Proportions in Observational Studies. Pharmaceutical Statistics. 2011;10(2):150–161. doi:10.1002/pst.433).

3. (Ming K, Rosenbaum PR. Substantial Gains in Bias Reduction from Matching with a Variable Number of Controls. Biometrics. 2000;56(1):118–124. doi:10.1111/j.0006‑341X.2000.00118.x; Rosenbaum PR. Observational Studies. 2nd ed. Springer; 2002).

4. (Niknam BA, Zubizarreta JR. Using Cardinality Matching to Design Balanced and Representative Samples for Observational Studies. JAMA. 2022;327(2):173–174. doi:10.1001/jama.2021.20555; Zubizarreta JR. Matching for Balance, Pairing for Heterogeneity in an Observational Study. Supplementary materials, 2014).

5. Stuart EA. Matching Methods for Causal Inference: A Review and a Look Forward. Statistical Science. 2010;25(1):1–21. doi:10.1214/09‑STS313.

6. Quan H, Sundararajan V, Halfon P, et al. Coding Algorithms for Defining Comorbidities in ICD‑9‑CM and ICD‑10 Administrative Data. Medical Care. 2005;43(11):1130–1139. doi:10.1097/01.mlr.0000182534.19832.83.

7. Quan H, Sundararajan V, Halfon P, et al. Coding algorithms for defining comorbidities in ICD-9-CM and ICD-10 administrative data. Medical Care. 2005;43(11):1130-1139. doi:10.1097/01.mlr.0000182534.19832.83.

8. Rosenbaum PR, Rubin DB. The central role of the propensity score in observational studies for causal effects. Biometrika. 1983;70:41-55).

9. Stuart EA. Matching methods for causal inference: a review and a look forward. Statistical Science. 2010;25:1-21. doi:10.1214/09-STS313

10. Statistics in Medicine. 2009;28:3083-3107

**Supplemental Tables**

**Table S1: Sleep-related Utilization Codes: TCRF and Medically Managed Cohorts**

| **Category** | **Code System** | **Code** | **Label** |
| --- | --- | --- | --- |
| **Sleep Study** | CPT/HCPCS | 95810 | Polysomnography (≥6 hrs) |
| **Sleep Study** | CPT/HCPCS | 95811 | Polysomnography with CPAP titration |
| **Sleep Study** | CPT/HCPCS | G0398 | Home sleep apnea test (Type II) |
| **Sleep Study** | CPT/HCPCS | G0399 | Home sleep apnea test (Type III) |
| **Sleep Study** | CPT/HCPCS | G0400 | Home sleep apnea test (Type IV) |
| **OSA Procedure** | CPT | 42975 | Pharyngoplasty for OSA |
| **Sleep Med** | Generic |  | zolpidem |
| **Sleep Med** | Generic |  | eszopiclone |
| **Sleep Med** | Generic |  | zaleplon |
| **Sleep Med** | Generic |  | temazepam |
| **Sleep Med** | Generic |  | triazolam |
| **Sleep Med** | Generic |  | lorazepam |
| **Sleep Med** | Generic |  | ramelteon |
| **Sleep Med** | Generic |  | suvorexant |
| **Sleep Med** | Generic |  | lemborexant |
| **Sleep Med** | Generic |  | daridorexant |
| **Sleep Med** | Generic |  | trazodone |
| **Sleep Med** | Generic |  | doxepin |

Abbreviations: PY, patient‑years; IRR, incidence rate ratio; CI, confidence interval; CPAP, continuous positive airway pressure; OSA, obstructive sleep apnea; PSG, polysomnography.

**Table S2: ENT Procedure Utilization by CPT Code Pre- and Post-Index TCRF**

| **CPT Code** | **ENT Procedure Type** | **Prev (%) (Pre)** | **Prev (%) (Post)** | **Rate/100 PY (Pre)** | **Rate/100 PY (Post)** | **Rate Ratio (95% CI), p** |
| --- | --- | --- | --- | --- | --- | --- |
| **00160** | Anesthesia for nose/sinus surgery | 6.30 | 1.66 | 7.06 | 2.85 | 0.40 (0.35–0.47), p<0.001 |
| **30130** | Excision inferior turbinate, partial/complete | 0.92 | 0.20 | 0.96 | 0.42 | 0.44 (0.31–0.63), p<0.001 |
| **30140** | Submucous resection inferior turbinate | 7.73 | 2.08 | 12.92 | 5.44 | 0.42 (0.38–0.47), p<0.001 |
| **30520** | Septoplasty/submucous resection | 7.80 | 2.43 | 10.13 | 5.12 | 0.50 (0.45–0.56), p<0.001 |
| **31240** | Nasal/sinus endoscopy, concha bullosa resection | 0.94 | 0.19 | 1.17 | 0.37 | 0.31 (0.21–0.46), p<0.001 |
| **31242** | Nasal/sinus endoscopy, surgical; destruction by radiofrequency ablation, posterior nasal nerve | 0.14 | 0.60 | 0.10 | 0.85 | 8.22 (4.71–14.33), p<0.001 |
| **31254** | Nasal/sinus endoscopy, ethmoidectomy, anterior | 1.39 | 0.45 | 1.65 | 0.74 | 0.45 (0.34–0.59), p<0.001 |
| **31256** | Nasal/sinus endoscopy, maxillary antrostomy | 0.96 | 0.34 | 1.57 | 0.93 | 0.59 (0.46–0.77), p<0.001 |
| **31267** | Endoscopic maxillary antrostomy with tissue removal | 2.19 | 0.57 | 3.53 | 1.44 | 0.41 (0.33–0.50), p<0.001 |
| **31276** | Endoscopic frontal sinusotomy | 1.26 | 0.36 | 1.92 | 0.86 | 0.45 (0.35–0.58), p<0.001 |
| **31295** | Endoscopic balloon dilation frontal sinus | 3.06 | 1.43 | 2.67 | 2.03 | 0.76 (0.63–0.91), p=0.003 |
| **31296** | Endoscopic balloon dilation maxillary sinus | 0.79 | 0.27 | 0.71 | 0.50 | 0.70 (0.49–1.00), p=0.049 |
| **31297** | Endoscopic balloon dilation sphenoid sinus | 0.24 | 0.12 | 0.28 | 0.18 | 0.64 (0.35–1.16), p=0.143 |
| **31298** | Endoscopic balloon dilation ≥2 sinuses | 1.85 | 1.06 | 1.60 | 1.43 | 0.89 (0.72–1.11), p=0.311 |
| **42826** | Tonsillectomy, primary/adult ≥12 yrs | 0.17 | 0.12 | 0.21 | 0.28 | 1.33 (0.78–2.26), p=0.301 |
| **42975** | Pharyngoplasty for sleep apnea (UPPP, lateral expansion) | 0.68 | 0.41 | 0.83 | 0.86 | 1.04 (0.78–1.39), p=0.785 |

**Table S3. Fragility and Durability Trimming Results for Evaluation and Management Visit Categories: TCRF Cohort**

| **E&M Category** | **Baseline**  **RR^1^ (95% CI)** | **1% trim**  **RR (95% CI)** | **5% trim**  **RR (95% CI)** | **% Post index claims from top 1% trimmed group (95% CI)** | **Classification** |
| --- | --- | --- | --- | --- | --- |
| **TCRF Cohort** | | | | | |
| Office/Outpatient | 0.92 (0.91–0.93) | 0.89 (0.88–0.90) | 0.83 (0.83–0.84) | 8.0% (7.5–8.5%) | Durable |
| Emergency Department | 0.86 (0.83–0.88) | 0.80 (0.77–0.82) | 0.67 (0.65–0.70) | 13.0% (12.3–13.7%) | Durable |
| Hospital Inpatient/Observation | 1.36 (1.33–1.40) | 1.13 (1.09–1.16) | 0.76 (0.74–0.79) | 48.0% (47.0–49.0%) | Fragile |
| Preventive Care | 0.76 (0.73–0.79) | 0.72 (0.69–0.75) | 0.65 (0.62–0.67) | 10.0% (9.4–10.6%) | Durable |
| Telemedicine/Remote | 0.73 (0.69–0.78) | 0.60 (0.56–0.63) | 0.44 (0.41–0.47) | 12.0% (11.4–12.6%) | Durable |
| Nursing Facility | 1.79 (1.64–1.96) | 1.66 (1.51–1.82) | 1.18 (1.06–1.31) | 37.0% (36.1–37.9%) | Fragile |
| Home/Residence | 0.81 (0.72–0.92) | 0.74 (0.65–0.84) | 0.58 (0.50–0.67) | 12.4% (8.9–15.9%) | Durable |
| Critical Care | 1.69 (1.53–1.86) | 1.46 (1.32–1.61) | 1.00 (0.89–1.12) | 44.0% (43.0–45.0%) | Fragile |
| **Medically Managed Cohort^2^** | | | | | |
| Office/Outpatient | 1.08 (1.08–1.09) | 1.06 (1.05–1.07) | 1.04 (1.03–1.05) | 7.8% (6.3–9.3%) | Durable |
| Emergency Department | 1.10 (1.09–1.11) | 0.99 (0.98–1.00) | 0.96 (0.95–0.97) | 14.7% (12.7–16.6%) | Fragile |
| Hospital Inpatient/Observation | 1.79 (1.77–1.80) | 0.29 (0.28–0.30) | 0.29 (0.28–0.30) | 8.1% (6.6–9.6%) | Durable |
| Preventive Care | 0.92 (0.90–0.93) | 0.89 (0.88–0.90) | 0.84 (0.83–0.85) | 9.9% (8.2–11.6%) | Durable |
| Telemedicine/Remote | 1.33 (1.30–1.36) | 1.27 (1.24–1.30) | 1.19 (1.16–1.22) | 5.2% (4.0–6.5%) | Fragile |
| Nursing Facility | 2.11 (2.06–2.16) | 1.94 (1.89–1.99) | 1.62 (1.57–1.67) | 9.4% (7.8–11.0%) | Fragile |
| Home/Residence | 1.60 (1.54–1.67) | 1.52 (1.46–1.58) | 1.36 (1.31–1.41) | 15.6% (13.6–17.6%) | Durable |
| Critical Care | 2.18 (2.11–2.26) | 1.97 (1.90–2.04) | 1.64 (1.58–1.70) | 13.1% (11.2–14.9%) | Fragile |

^1^Rate ratios are shown at baseline and after trimming 1% and 5% of patients, with classification of effects as fragile or durable.

^2^ ATT weighted

**Table S4: Sleep-related Healthcare Resource Utilization by CPT/HCPCS Codes: TCRF Cohort**

| Code | Label | Prev (%) (Pre) | Prev (%) (Post) | Rate/100 PY (Pre) | Rate/100 PY (Post) | Rate Ratio (95% CI), p |
| --- | --- | --- | --- | --- | --- | --- |
| 95810 | Polysomnography (≥6 hrs) | 4.47 | 1.88 | 3.39 | 1.4 | 0.41 (0.36–0.48), p<0.001 |
| 95811 | Polysomnography with CPAP titration | 3.66 | 1.7 | 3.08 | 1.27 | 0.41 (0.36–0.48), p<0.001 |
| G0398 | Home sleep apnea test (Type II) | 0.07 | 0.04 | 0.03 | 0.02 | 0.57 (0.17–1.95), p=0.372 |
| G0399 | Home sleep apnea test (Type III) | 2.37 | 0.74 | 1.74 | 0.48 | 0.28 (0.22–0.35), p<0.001 |
| G0400 | Home sleep apnea test (Type IV) | 0.33 | 0.15 | 0.22 | 0.09 | 0.40 (0.23–0.69), p=0.001 |
| 42975 | Pharyngoplasty for OSA | 0.68 | 0.41 | 0.59 | 0.36 | 0.61 (0.45–0.81), p<0.001 |
| Obstructive Sleep Apnea Procedures | | | | | | |
| 42975 | Pharyngoplasty for OSA | 0.68 | 0.41 | 0.59 | 0.36 | 0.61 (0.45–0.81), p<0.001 |

Abbreviations: PY, patient‑years; IRR, incidence rate ratio; CI, confidence interval; CPAP, continuous positive airway pressure; OSA, obstructive sleep apnea; PSG, polysomnography.

**Table S5. Sleep Medications (all Fills/Refills): TCRF cohort**

| **Medication** | **Prevalence (%) Pre-Index** | **Prevalence (%) Post-Index** | **Rate/100 PY Pre-Index** | **Rate/100 PY Post-Index** | **Rate Ratios (95%, p)** |
| --- | --- | --- | --- | --- | --- |
| **daridorexant** | 0.0 | 0.0 | 0.0 | 0.0 | 1.00 (0.02–50.40), p=1.000 |
| **doxepin** | 0.0 | 0.0 | 0.0 | 0.0 | 1.00 (0.02–50.40), p=1.000 |
| **eszopiclone** | 1.28 | 0.92 | 3.95 | 2.81 | 0.71 (0.64–0.79), p<0.001 |
| **lemborexant** | 0.22 | 0.09 | 0.24 | 0.14 | 0.59 (0.37–0.94), p=0.025 |
| **lorazepam** | 5.41 | 3.41 | 11.4 | 7.04 | 0.62 (0.58–0.66), p<0.001 |
| **ramelteon** | 0.36 | 0.24 | 0.63 | 0.34 | 0.54 (0.40–0.72), p<0.001 |
| **suvorexant** | 0.28 | 0.21 | 0.48 | 0.46 | 0.96 (0.72–1.27), p=0.773 |
| **temazepam** | 0.95 | 0.65 | 3.75 | 1.97 | 0.52 (0.47–0.59), p<0.001 |
| **trazodone** | 0.0 | 0.0 | 0.0 | 0.0 | 1.00 (0.02–50.40), p=1.000 |
| **triazolam** | 1.56 | 0.47 | 1.09 | 0.37 | 0.34 (0.26–0.44), p<0.001 |
| **zaleplon** | 0.3 | 0.22 | 0.81 | 0.66 | 0.81 (0.64–1.01), p=0.065 |
| **zolpidem** | 0.0 | 0.0 | 0.0 | 0.0 | 1.00 (0.02–50.40), p=1.000 |

## **Table S6. Top 5 Diagnoses Overall vs Top 1% Utilizers**

| **E/M Category** | **Overall and Top 1% Event Rank** | **ICD-10 Code** | **ICD-10 Description** | **Overall % Events** | **Top 1% Events** |
| --- | --- | --- | --- | --- | --- |
| **Critical Care** | 1 | J910 | Malignant pleural effusion | -- | 17.0 |
|  |  | J9601 | Acute respiratory failure with hypoxia | 9.4 | 8.0 |
|  | 2 | A419 | Sepsis, unspecified organism | 5.0 | -- |
|  |  | E781 | Pure hyperglyceridemia | -- | 12.5 |
|  | 3 | G40901 | Epilepsy, unspecified, not intractable, with status epilepticus | -- | 11.4 |
|  |  | I4891 | Unspecified atrial fibrillation | 5.0 | -- |
|  | 4 | I639 | Cerebral infarction, unspecified | 3.7 | -- |
|  |  | T8509XA | Mechanical complications of ventricular intracranial shunt, initial | -- | 10.2 |
|  | 5 | I2699 | Other pulmonary embolism without acute cor pulmonale | 2.1 | -- |
| **Emergency Department** | 1 | R0789 | Other chest pain | 3.5 | 3.0 |
|  |  | R1084 | Generalized abdominal pain | -- | 3.4 |
|  | 2 | R079 | Chest pain, unspecified | 2.3 | 2.9 |
|  |  | U071 | U071 | -- | 3.4 |
|  | 3 | R42 | Dizziness and giddiness | 2.1 | -- |
|  | 4 | R040 | Epistaxis | 1.8 | -- |
|  | 5 | M5450 | M5450 | 1.7 | -- |
|  |  | R109 | Unspecified abdominal pain | -- | 2.4 |
| **Home/Residence** | 1 | F4322 | Adjustment disorder with anxiety | -- | 18.0 |
|  |  | I10 | Essential (primary) hypertension | 9.6 | -- |
|  | 2 | I480 | Paroxysmal atrial fibrillation | -- | 15.4 |
|  |  | Z0000 | Encounter for general adult medical exam w/o abnormal findings | 8.2 | -- |
|  | 3 | E785 | Hyperlipidemia, unspecified | 4.1 | -- |
|  |  | I130 | Hypertrophy heart & chronic kidney disease w heart fail and stage 1-4/unspecified chronic kidney | -- | 12.8 |
|  | 4 | E119 | Type 2 diabetes mellitus without complications | 3.0 | -- |
|  |  | G1221 | Amyotrophic lateral sclerosis | -- | 7.7 |
|  | 5  5 | E1122 | Type 2 diabetes mellitus w diabetic chronic kidney disease | 2.5 | -- |
|  |  | J9611 | Chronic respiratory failure with hypoxia | -- | 7.7 |
| **Hospital Inpatient/Observation** | 1 | N179 | Acute kidney failure, unspecified | 4.4 | 10.1 |
|  | 2 | A419 | Sepsis, unspecified organism | 2.6 | -- |
|  |  | J189 | Pneumonia, unspecified organism | -- | 4.3 |
|  | 3 | G893 | Neoplasm related pain (acute) (chronic) | -- | 3.3 |
|  |  | I10 | Essential (primary) hypertension | 2.4 | -- |
|  | 4 | E46 | Unspecified protein-calorie malnutrition | -- | 3.0 |
|  |  | I4891 | Unspecified atrial fibrillation | 2.3 | -- |
|  | 5 | J9601 | Acute respiratory failure with hypoxia | 2.2 | -- |
|  |  | R0602 | Shortness of breath | -- | 3.0 |
| **Nursing Facility** | 1 | M6281 | Muscle weakness (generalized) | 8.3 | 19.7 |
|  | 2 | I10 | Essential (primary) hypertension | 6.7 | 15.8 |
|  |  | I69351 | Hemiplegia following cerebral infarction affecting right dominant side | -- | 17.1 |
|  | 3 | J449 | Chronic obstructive pulmonary disease, unspecified | 4.1 | -- |
|  | 4 | E119 | Type 2 diabetes mellitus without complications | -- | 10.5 |
|  |  | J189 | Pneumonia, unspecified organism | 3.0 | -- |
|  | 5 | I6389 | I6389 | -- | 9.2 |
|  |  | S42021D | Displaced fracture of shaft of right clavicle, subs for fracture with routine healing | 2.6 | -- |
| **Office/Outpatient** | 1 | E291 | Testicular hypofunction | -- | 2.8 |
|  |  | I10 | Essential (primary) hypertension | 3.6 | 2.4 |
|  | 2 | G4733 | Obstructive sleep apnea (adult) (pediatric) | 2.3 | -- |
|  | 3 | J3489 | Other specified disorders of nose and nasal sinuses | 1.6 | -- |
|  |  | M542 | Cervicalgia | -- | 1.7 |
|  | 4 | M5416 | Radiculopathy, lumbar region | -- | 1.6 |
|  |  | R0981 | Nasal congestion | 1.4 | -- |
|  | 5 | E119 | Type 2 diabetes mellitus without complications | 1.0 | -- |
|  |  | F331 | Major depressive disorder, recurrent, moderate | -- | 1.5 |
| **Preventive Care** | 1 | Z0000 | Encounter for general adult medical exam w/o abnormal findings | 56.7 | 42.1 |
|  | 2 | Z01419 | Encounter for gyn exam (general) (routine) w/o abnormal findings | 17.3 | 29.2 |
|  | 3 | Z0001 | Encounter for general adult medical exam w abnormal findings | 13.6 | 11.4 |
|  | 4 | Z01411 | Encounter for gynecologic exam (general) (routine) w abnormal findings | 2.2 | 3.2 |
|  | 5 | Z23 | Encounter for immunization | 0.9 | 1.2 |
| **Telemedicine/Remote** | 1 | I10 | Essential (primary) hypertension | 26.3 | 64.7 |
|  | 2 | G4733 | Obstructive sleep apnea (adult) (pediatric) | 5.3 |  |
|  |  | J4530 | Mild persistent asthma, uncomplicated | 2.2 | 11.4 |
|  | 3 | I129 | Hypertensive chronic kidney disease w stg 1-4/unspecified chronic kidney | -- | 6.9 |
|  | 4 | E7800 | E7800 | -- | 6.2 |
|  |  | E785 | Hyperlipidemia, unspecified | 1.9 | -- |
|  | 5 | G894 | Chronic pain syndrome | -- | 4.2 |
|  |  | U071 | U071 | 1.5 | -- |

**Table S7. Baseline characteristics of the TCRF and MM cohorts before and after ATT weighting: TCRF Cohort**

| Characteristic^1^ | TCRF(N=10,206) | MM (Unweighted N=50,766) | MM 10206  Weighted^2^ | \|SMD\| Unweighted | \|SMD\| Weighted |
| --- | --- | --- | --- | --- | --- |
| Age, mean (SD) | 60.9 (17.4) | 55.7 (17.1)^3^ | 60.2 (16.3) | 0.224 | 0.046 |
| Female, %3 | 46.6 | 47.2 | 46 | 0.799 | 0.022 |
| Male, % | 51.9 | 52.8 | 52 | 0.874 | 0.051 |
| Unknown sex, % | 1.5 | 0.0 | 0.0 | 0.070 | 0.172 |
| Charlson, mean (SD) | 1.85 (1.96) | 1.90 (2.11) | 2.32 (2.28) | 0.03 | 0.22 |
| Charlson ≥1, % | 67.9 | 65.1 | 69.8 | 0.06 | 0.04 |
| ^1^Abbreviations: SD, standard deviation; SMD, standardized mean difference; ESS, effective sample size after ATT weighting. ^2^Values for weighted MMs represent weighted means or proportions aligned to the TCRF cohort. SMDs are reported as absolute values, close to 0 indicate good balance; values <0.1 are generally considered acceptable.  ^3^Percentages are column percentages. ESS was calculated as (Σw)² / Σw², reflecting the information content of the weighted MM cohort.  ^4^ Effective sample size (ESS) after weighting is also presented to demonstrate preservation of analytic precision.  *Note:* Covariate balance targeted ENT disease‑state severity (pre‑index ENT costs/claims, spray intensity, NAO diagnosis frequency) ahead of general comorbidity to align the matched/weighted MM cohort with the treated NAO/NVC case‑mix. Charlson was retained with a ±25% caliper and monitored post‑weighting. | | | | | |

**Supplemental Figures**

**Figure S1.** Cost trends in TCRF and MM cohorts, comparing weighted and unweighted estimates. (95% CI; monthly dashed, smoothed solid)


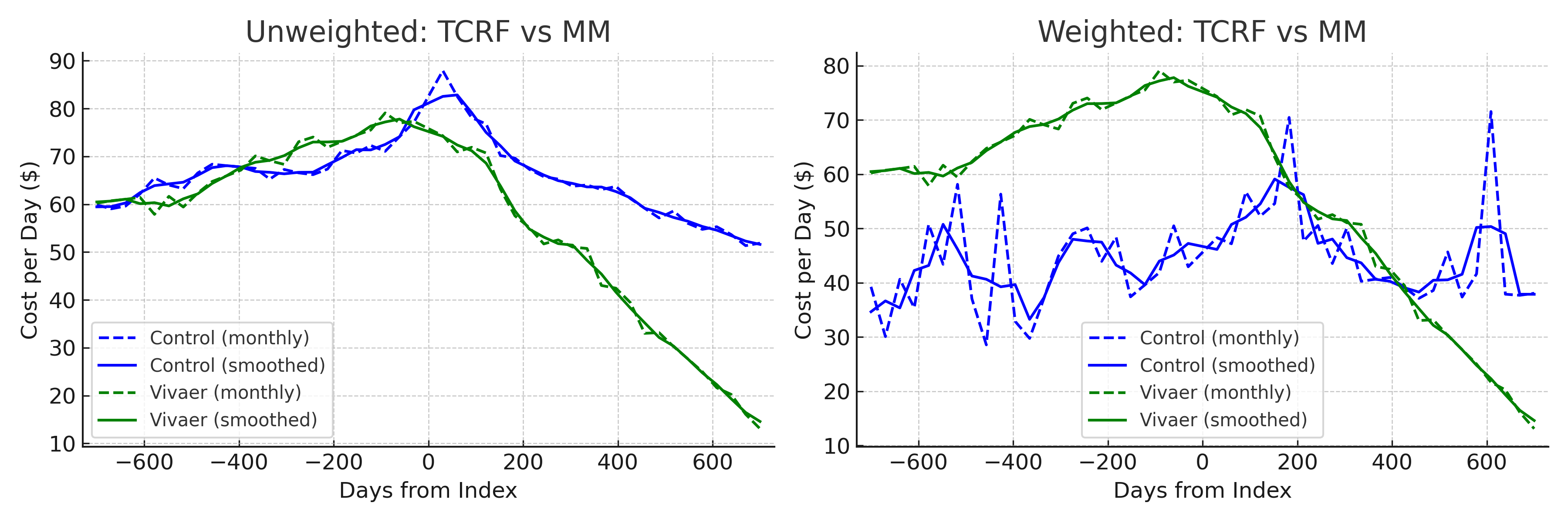


**Figure S2.** Average patient daily cost comparison of TCRF versus MM extending MM out to 48 months post-index.

**
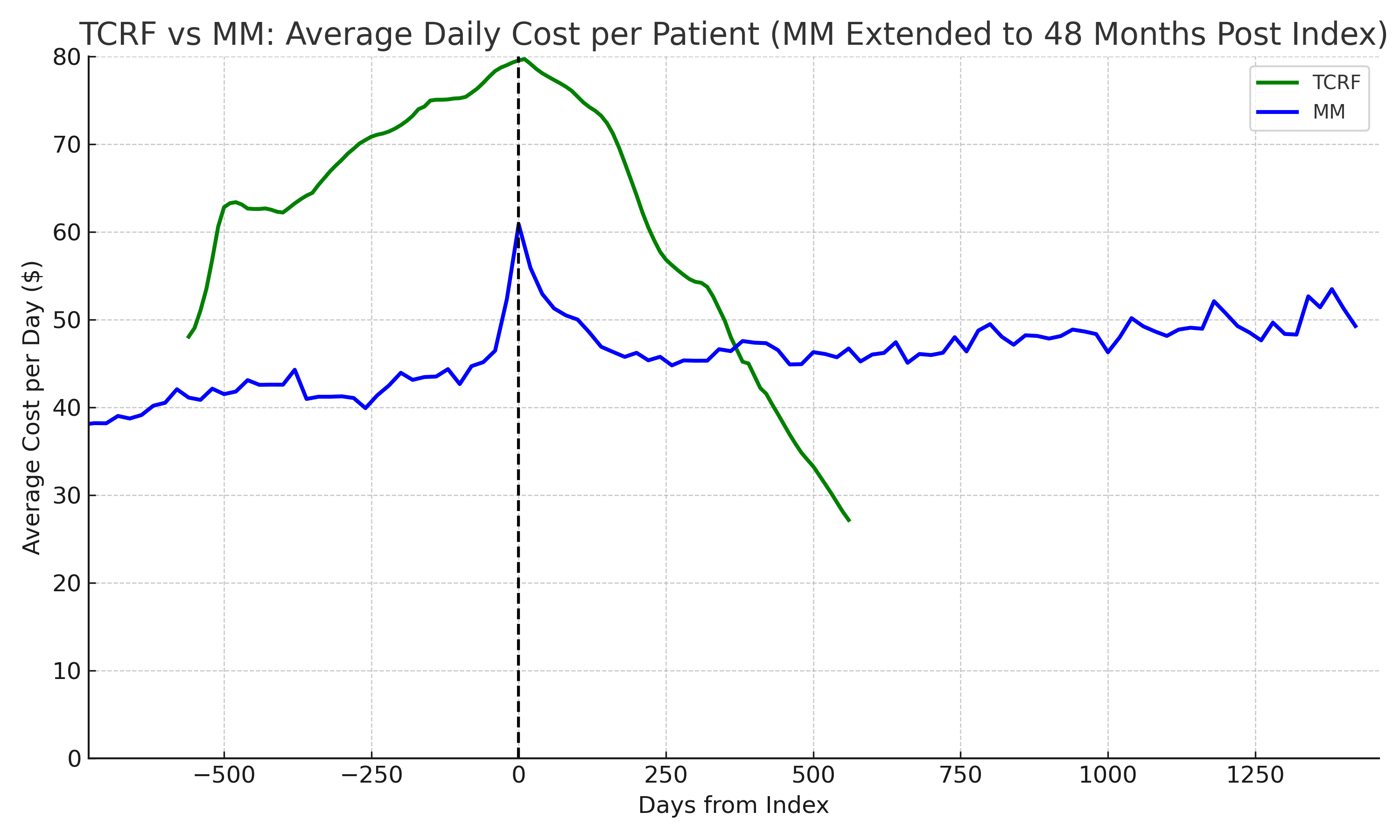
**

**Figure S3.** Fragility and durability trimming curves for E&M utilization categories. Panels display rate ratios across trimming thresholds with 95% confidence intervals.


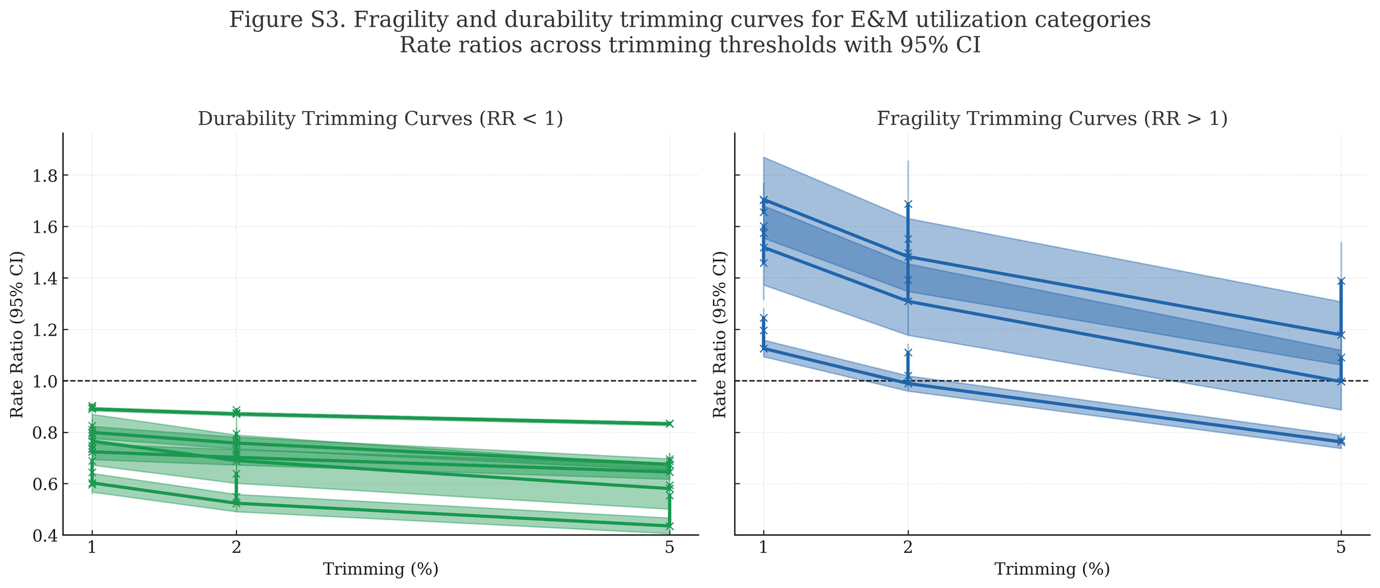


**Figure S4.** Robustness checks using winsorization (95th–99th percentile caps) and leave-k%-out resampling. Curves illustrate stability of key utilization findings under these sensitivity analyses. (blue=fragility, green=durability)

**
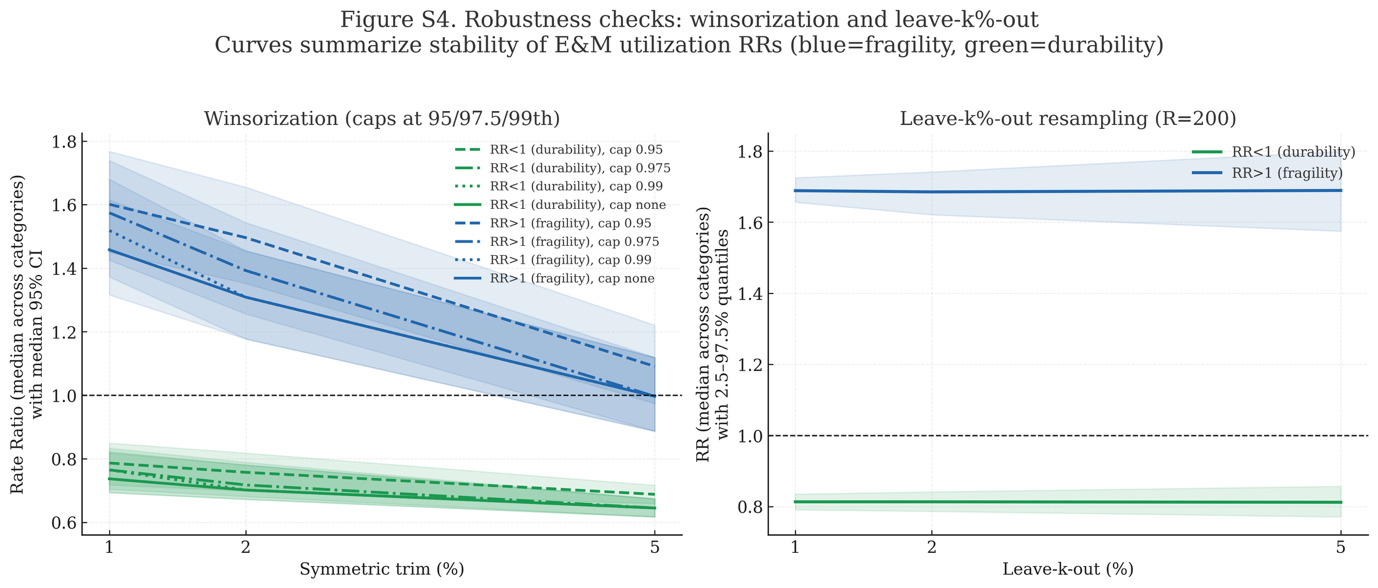
**
